# Membrane alterations induced by nonstructural proteins of human norovirus

**DOI:** 10.1371/journal.ppat.1006705

**Published:** 2017-10-27

**Authors:** Sylvie Y. Doerflinger, Mirko Cortese, Inés Romero-Brey, Zach Menne, Thibault Tubiana, Christian Schenk, Peter A. White, Ralf Bartenschlager, Stéphane Bressanelli, Grant S. Hansman, Volker Lohmann

**Affiliations:** 1 Department of Infectious Diseases, Virology, Heidelberg University, Heidelberg, Germany; 2 Schaller Research Group at the University of Heidelberg and the DKFZ, Heidelberg, Germany; 3 Department of Infectious Diseases, Molecular Virology, Heidelberg University, Im Neuenheimer Feld 345, Heidelberg, Germany; 4 Institute for Integrative Biology of the Cell (I2BC), CEA, CNRS, Univ Paris Sud, Université Paris-Saclay, Gif sur Yvette cedex, France; 5 School of Biotechnology and Biomolecular Sciences, Faculty of Science, University of New South Wales, Sydney, Australia; 6 German Center for Infection Research (DZIF), Heidelberg University, Heidelberg, Germany; Stanford University School of Medicine, UNITED STATES

## Abstract

Human noroviruses (huNoV) are the most frequent cause of non-bacterial acute gastroenteritis worldwide, particularly genogroup II genotype 4 (GII.4) variants. The viral nonstructural (NS) proteins encoded by the ORF1 polyprotein induce vesical clusters harboring the viral replication sites. Little is known so far about the ultrastructure of these replication organelles or the contribution of individual NS proteins to their biogenesis. We compared the ultrastructural changes induced by expression of norovirus ORF1 polyproteins with those induced upon infection with murine norovirus (MNV). Characteristic membrane alterations induced by ORF1 expression resembled those found in MNV infected cells, consisting of vesicle accumulations likely built from the endoplasmic reticulum (ER) which included single membrane vesicles (SMVs), double membrane vesicles (DMVs) and multi membrane vesicles (MMVs). In-depth analysis using electron tomography suggested that MMVs originate through the enwrapping of SMVs with tubular structures similar to mechanisms reported for picornaviruses. Expression of GII.4 NS1-2, NS3 and NS4 fused to GFP revealed distinct membrane alterations when analyzed by correlative light and electron microscopy. Expression of NS1-2 induced proliferation of smooth ER membranes forming long tubular structures that were affected by mutations in the active center of the putative NS1-2 hydrolase domain. NS3 was associated with ER membranes around lipid droplets (LDs) and induced the formation of convoluted membranes, which were even more pronounced in case of NS4. Interestingly, NS4 was the only GII.4 protein capable of inducing SMV and DMV formation when expressed individually. Our work provides the first ultrastructural analysis of norovirus GII.4 induced vesicle clusters and suggests that their morphology and biogenesis is most similar to picornaviruses. We further identified NS4 as a key factor in the formation of membrane alterations of huNoV and provide models of the putative membrane topologies of NS1-2, NS3 and NS4 to guide future studies.

## Author summary

Positive-strand RNA viruses induce membrane alterations harboring the viral replication complexes. In the case of human noroviruses (huNoV), the major cause of acute viral gastroenteritis, these are induced by the ORF1 polyprotein, which is post-translationally processed into the functional nonstructural (NS) proteins. Partly due to the lack of efficient cell culture models, little is known so far about membrane alterations induced by huNoV belonging to the most clinically relevant genogroup II, genotype 4 (GII.4), nor about the function of individual NS proteins in their formation. We therefore expressed ORF1 proteins of GII.4 and individual NS proteins in cells to study their contribution to viral replication complex formation. Expression of ORF1 proteins of GII.4 induced vesicular membrane alterations comparable to those found in infected cells and similar to picornaviruses and hepatitis C virus (HCV). GII.4 NS1-2, NS3 and NS4 are contributing to viral membrane alterations. Our work provides new insights into their function in huNoV induced replication complex formation while identifying NS4 as the most important single determinant. This knowledge might provide novel attractive targets for future therapies inhibiting the formation of the membranous viral replication complex, as exemplified by the efficacy of HCV NS5A inhibitors.

## Introduction

Human noroviruses (huNoV) are the most frequent causative agent of acute gastroenteritis worldwide, responsible for over 30% of all cases, subsequently resulting in over 200,000 deaths per annum [1]. Still, no vaccine or specific antiviral therapy is available to counteract huNoV infections. Noroviruses are divided into seven different genogroups (GI-GVII) and further subdivided into numerous genotypes [2]. Noroviruses grouped into GI, GII and GIV mainly infect humans but also other species, while GV infects mice. The GII genotype 4 (GII.4) cause the majority of infections with novel outbreak strains emerging every 2–3 years, likely in a response to an immunological pressure of herd immunity [3–5].

Noroviruses belong to the *Caliciviridae* family and have a positive-sense single-stranded RNA genome of approximately 7.5 kilobases (kb) (reviewed in [6]). The huNoV genome contains three open reading frames (ORFs), where ORF1 encodes the non-structural proteins (NS1-7) involved in viral replication, ORF 2 encodes the capsid protein and ORF3 encodes a small structural protein. Murine noroviruses (MNV) additionally encode an ORF4 from an alternative reading frame located in ORF2, termed virulence factor 1 (VF1), involved in antagonism of the host innate immune response [7]. The 5’ end of the genome contains a short 5 nucleotide untranslated region (UTR) and the 3’end contains a short UTR and poly-A tail (reviewed in [8]). The norovirus genome is covalently linked at the 5’end with the viral protein VPg (also termed NS5). ORF1 is translated from the full-length genomic RNA, whereas ORF2, ORF3, and ORF4 are mainly translated from a VPg linked subgenomic RNA (reviewed in [8]).

ORF1 encodes a large, approximately 200 kDa, polyprotein that is processed by the viral protease NS6, giving rise to 6 mature nonstructural proteins involved in viral replication and several precursor proteins with potentially additional, yet poorly defined functions (reviewed in [8]). The function of the most N-terminal protein (termed NS1-2 or p48) is unclear. huNoV NS1-2 varies in size (approximately 40–48 kDa) and contains an N-terminal disordered region and a C-terminal predicted trans-membrane domain [9]. The central domain further shows homology to the NlpC/p60 superfamily of enzymes, with diverse hydrolase functions [10]. Genogroup I NS1-2 has been shown to localize to the Golgi complex and induce Golgi disassembly, dependent upon the C-terminal hydrophobic region [11]. MNV NS1/2 contains 2 sites cleaved by murine caspase 3 and has been shown to localize to the endoplasmic reticulum (ER) upon transient expression [12,13]. NS3 (also termed NTPase, 2C-like and p41) has been demonstrated to function as an NTPase *in vitro* for GI [14]. NS3 has also been shown to co-localize with double stranded RNA (dsRNA) during MNV infection [15]. NS4 (also called p20, p22 or 3A-like) function remains unclear, although NS4 has been demonstrated to disrupt ER to Golgi trafficking resulting in Golgi disassembly during norovirus replication [16]. NS4 has also been shown to inhibit actin cytoskeleton remodeling in an epithelial cell line upon transient expression [17]. Upon MNV infection, NS4 was shown to localize to the replication complex [15], and upon transient expression shown to localize to endosomes [13]. NS5 is linked to the 5’ end of the genome and plays an integral role in the initiation of translation through its interaction with eukaryotic initiation factors and likely primes genome and subgenomic RNA synthesis [18]. The viral protease NS6 (also called Pro or 3C-like) is a well characterized cysteine protease and responsible for the cleavage and processing of the viral ORF1 polyprotein [12,19]. Lastly, NS7 (also called RdRp) functions as the RNA dependent RNA polymerase in viral replication and transcription of subgenomic RNAs [20,21]).

Membrane rearrangements play a key role in the establishment of viral replication complexes for positive strand RNA viruses. In principle these membrane alterations can be subdivided into two morphotypes (reviewed in [22,23]). First, the “invagination type” consists of single membrane invaginations of a donor membrane which stay connected to the cytoplasm via a pore and are represented by alphaviruses and flaviviruses. Viral replication takes place inside these vesicles and the viral RNA contributes to their morphology [24], with the exception of Brome mosaic virus 1a protein which generates spherules in absence of RNA replication [25]. Second, the “DMV-type” consists of vesicular and tubular membrane rearrangements wrapped by one (single membrane vesicle, SMV), two (double membrane vesicles, DMVs) and multiple membranes (multi membrane vesicles, MMVs), induced by picornaviruses, coronaviruses and hepatitis C virus (HCV). Most of these structures have no visible connection to the cytoplasm and the functional significance of the different vesicle subtypes as well as the localization of the RNA synthesis machinery is still a matter of debate. However, these structures can typically be induced simply by expression of the replicase proteins in absence of RNA replication, exemplified by picornaviruses [26–29] and HCV [30–32]. Sole expression of individual nonstructural proteins already induces distinct membrane alterations, which are less complex than those derived from the polyprotein. Still, such studies have allowed the identification of those viral proteins contributing to the morphogenesis of viral replication sites and the unraveling of some of their functions [26–28,30,31,33–35]. In the case of HCV, virus induced membrane alterations have been identified as efficient drug targets for silibinin [36], direct acting antivirals like NS5A inhibitors [37] and host targeting drugs like cyclophilin inhibitors [38].

Our understanding of the ultrastructure of huNoV replication organelles is currently scarce, mostly due to the lack of efficient cell culture models [39]. A replicon model has been established in case of GI noroviruses [40], but no ultrastructural analysis is currently available. A plasmid driven GII.3 replicon model allows moderate RNA replication levels, but it remains difficult to dissect the contribution of protein expression and *bona fide* RNA replication in this system [41]. Recently, tremendous progress has been achieved in cultivating the more clinically relevant GII.4 strains in both B-cells [42] and enteric organoids [43], still neither of these models has yet been proven to allow ultrastructural studies. Therefore, most of our knowledge of norovirus induced membrane alterations has been obtained using the MNV model [44]. Previous studies showed accumulations of vesicles in the cytoplasm of infected macrophages consisting of single and double membrane vesicles, which have not been further characterized [44]. In addition, it has been shown that MNV induced vesicle clusters co-localize with all MNV NS proteins and with viral replication intermediates and that these extensive rearrangements of intracellular membranes are mainly derived from the secretory pathway, including ER, Golgi and endosomal membranes [15]. Furthermore, the MNV replication organelles seem tightly associated with the cytoskeleton, probably mediated by the main capsid protein [45]. Little is known so far about the contribution of individual nonstructural proteins to virus induced replication vesicles, but it is believed that NS1-2 and NS4 are the main drivers in this process due to their membrane association and comparison to picornavirus proteins (reviewed in [8]). In addition, NS3 is associated with membranes and recently has been shown to be associated with highly motile vesicular structures [13,46].

The current study aimed to investigate membrane alterations induced by clinically highly prevalent GII.4 using a transient expression system in Huh7 cell lines. Membrane structures induced by expression of the polyprotein of three important outbreak strains (Den Haag 2006, New Orleans 2009 and Sydney 2012) comprised SMV, DMV and MMV structures. We further observed that SMVs and DMVs were reminiscent of structures found upon MNV infection. The impact of individual GII.4 NS proteins on intracellular membranes was studied by correlative light and electron microscopy, allowing the localization of each protein within the cellular ultrastructural context. GII.4 NS1-2 induced membrane proliferation of the smooth ER, which was strikingly different from MNV NS1/2. NS3 was tightly associated with lipid droplets (LDs) and induced convoluted membranes. However, only NS4 expression was sufficient to induce SMV and DMV formation, much like the ability of HCV NS5A and poliovirus (PV) 3AB to induce DMVs.

## Results

### Expression of MNV ORF1 induces membrane rearrangements comparable to MNV infection

We aimed to study the determinants of membrane alterations induced by huNoV with a specific focus on clinically relevant GII.4 outbreak strains. We therefore wanted to exploit expression of ORF1 and of individual NS-proteins to assess the morphology of virus induced membrane alterations in Huh7 cells. We chose Huh7 cells for two reasons: first, Huh7 cells have been shown to support RNA replication of a human GI Norwalk replicon [40] and a plasmid based GII.3 replicon [41], suggesting that they are in principle permissive for huNoV. Second, Huh7 have been used to study membrane alterations for a variety of positive strand RNA viruses, including HCV, hepatitis A virus (HAV), coronaviruses and Dengue virus (reviewed in [22,23]), thereby facilitating the comparison of these structures among different virus groups.

**Fig 1:**
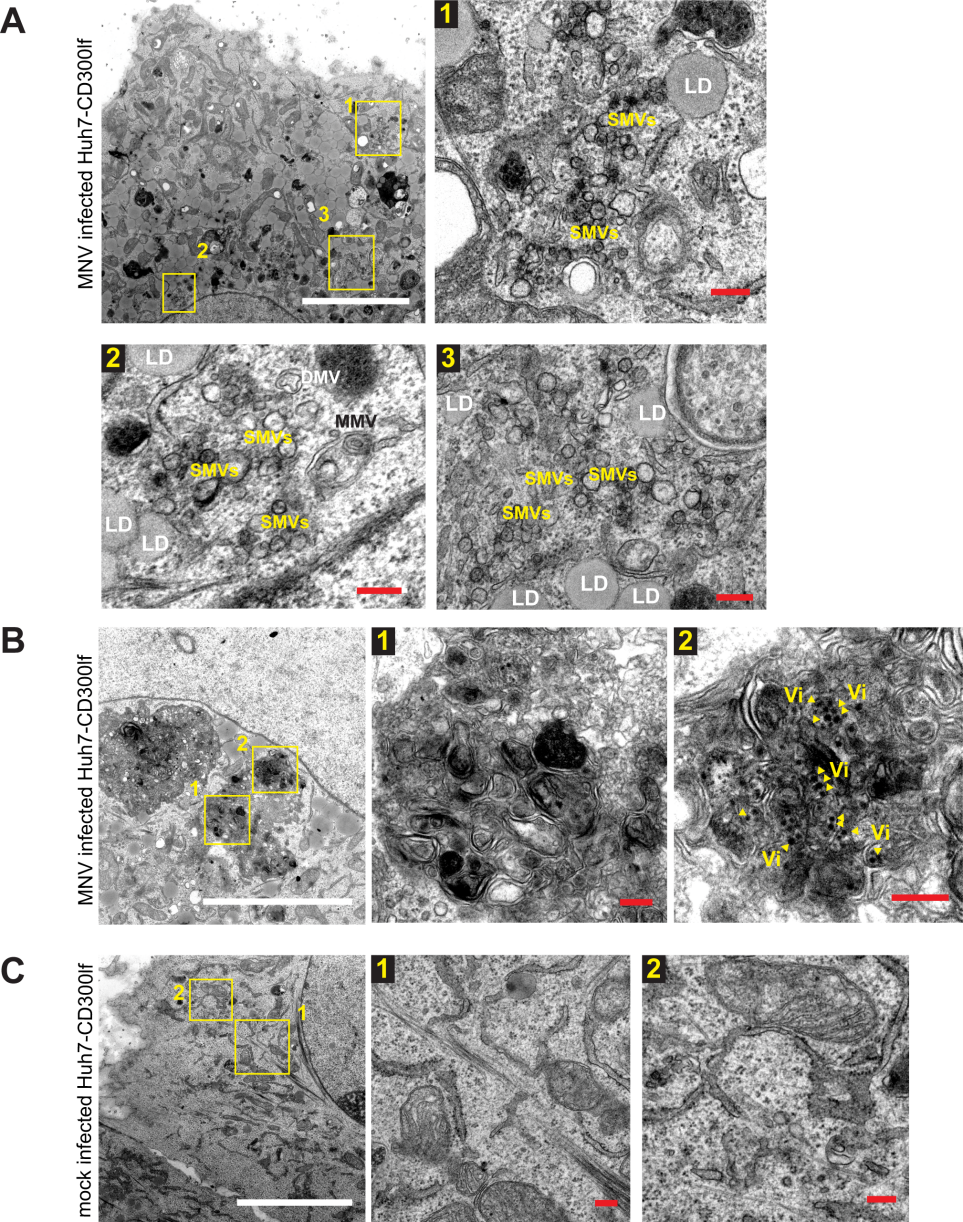
Ultrastructure of MNV infected Huh7-CD300lf cells. Huh7-CD300lf cells were infected (A, B) or mock infected (C) with MNV for 16 h (MOI = 1) before being chemically fixed and subjected to EM. Boxed and numbered areas are shown in higher magnification in subsequent panels. SMV, single membrane vesicle; DMV, double membrane vesicle; MMV, multi membrane vesicle; LD, lipid droplet. Note that SMVs are separated from the surrounding cytosol by a unique lipid bilayer. DMVs are delimited from the cytosol via two lipid bilayers and have a diameter below 300 nm. DMVs have normally a more electron-dense content than SMVs, likely due to engulfment of cytosolic content. MMVs contain more than 2 membranes. Yellow arrowheads indicate virions (Vi). White scale bars, 5 μm. Red scale bars, 200 nm.

**Fig 2:**
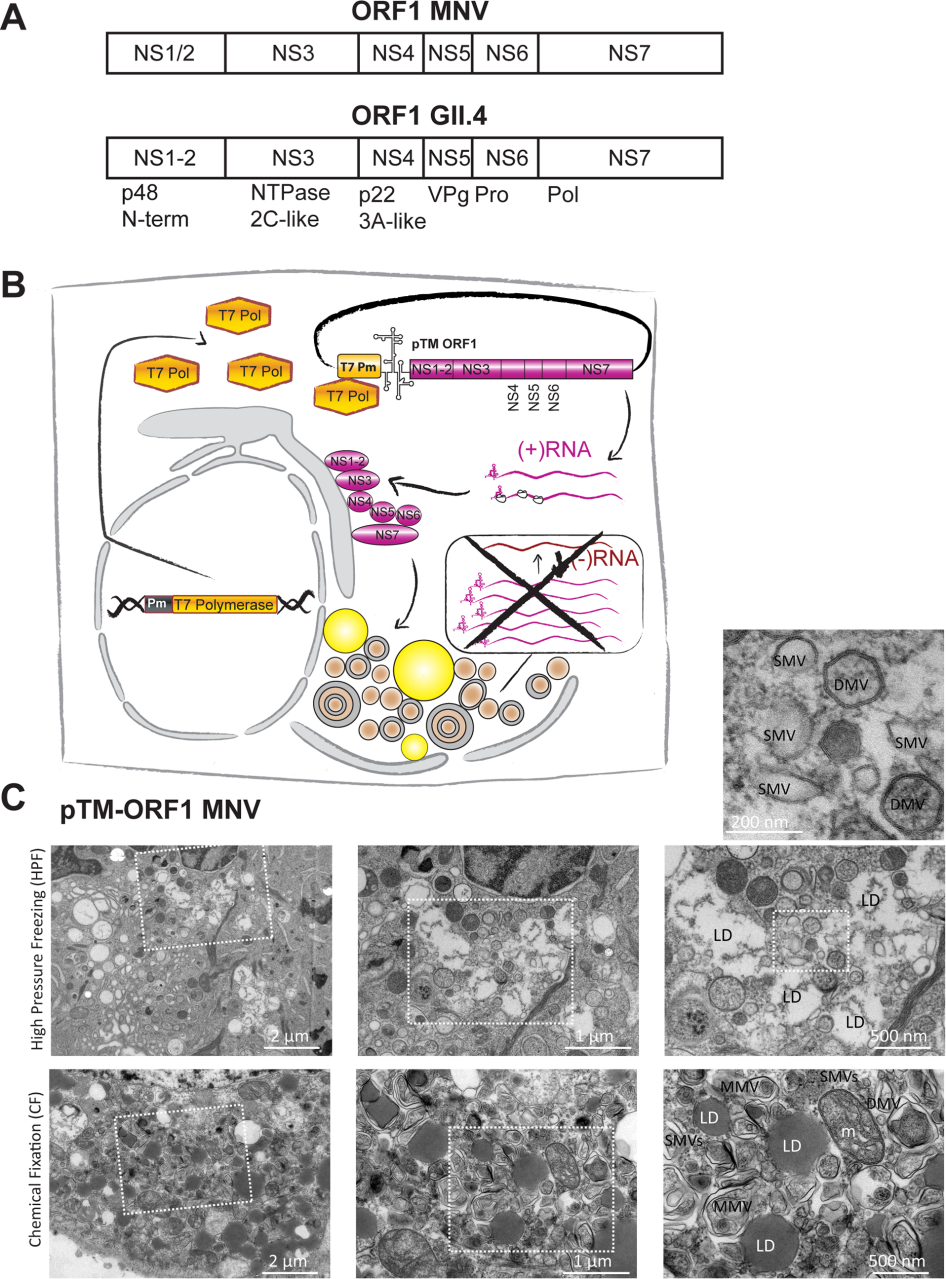
Expression model used in this study and ultrastructural analysis of membrane alterations induced by MNV ORF1 expression. (A) Schematic representation of the ORF1 polyprotein of MNV (top) and huNoV GII.4, indicating alternative nomenclature for the non-structural proteins (NS). The terminology in the boxes is used throughout this study. (B) Schematic representation of the protein expression model used in this study. Huh7 cells stably expressing T7-RNA polymerase are transfected with plasmids encoding ORF1 under transcriptional control of a T7 promoter and translational control of an EMCV IRES element, indicated by a secondary structure. Cytoplasmic expression of ORF1 proteins induces membrane alterations similar to infected cells in absence of RNA replication, indicated by a crossed box. (C) Huh7 T7 cells transfected with a construct expressing MNV ORF1 were processed for analysis by transmission EM (TEM) 20 h p.t. upon chemical fixation (CF) or high pressure freezing (HPF). Samples designated CF were grown on glass coverslips, fixed chemically with aldehydes, post-fixed with heavy metals, dehydrated with ethanol and resin embedded for further TEM analysis. Samples designated HPF were grown on sapphire discs, cryo-fixed by high pressure freezing, freeze substituted with heavy metals dissolved in acetone, dehydrated with acetone and resin embedded (reviewed in [97]). Representative images of both fixation techniques are shown as specified on the left. Magnified views of the boxed areas are shown to the right and on top. Single membrane vesicles (SMVs), double membrane vesicles (DMVs), multi-membrane vesicles (MMVs), lipid droplets (LDs) and mitochondria (m) are indicated in the high magnification views.

We first aimed to evaluate whether structures induced by ORF1 expression indeed resembled those found in infected cells using MNV as a model, ideally using the same cell type as intended for huNoV. We therefore generated Huh7-CD300lf cells stably expressing the MNV receptor [47,48] and verified that these cells were indeed permissive for MNV infection by demonstrating the presence of NS3 24h after infection (S1A and S1B Fig). In addition, MNV infected Huh7-CD300lf cells produced similar amounts of progeny virus compared to RAW 264.7 cells, albeit with slightly delayed kinetics (S1C Fig). In contrast, Huh7 cells lacking CD300lf failed to amplify the virus inoculum (S1C Fig). Therefore, ectopic expression of CD300lf rendered Huh7 cells fully permissive for MNV infection and supported the entire MNV replication cycle. Ultrastructural analysis of MNV infected Huh7-CD300lf revealed two major phenotypes not observed in uninfected cells (Fig 1): (1) Areas containing vesicles with a variety of shapes, sizes and types (Fig 1A). In addition to previously reported SMVs, more complex structures like DMVs and MMVs were found, often in proximity to lipid droplets (LD). This phenotype likely resembled an early replication phase described previously [44]. (2) A massive rearrangement of the entire endomembrane system consisting of complex structures, often associated with virions, and lacking an organized morphology was observed during what was likely a later stage of the replication cycle [44] (Fig 1B). We found similar phenotypes in RAW 264.7 cells (S2 Fig), which have been used in previous studies to characterize the ultrastructure of the MNV replication organelle [15,44], except that these cells lacked LDs (S2 Fig). We next analyzed whether similar structures were generated by expression of MNV ORF1. MNV ORF1 was expressed in Huh7 T7 cells under transcriptional control of the T7 promoter and translational control of an encephalomyocarditis virus internal ribosomal entry site (EMCV IRES), allowing efficient cytoplasmic expression of proteins of interest in the presence of T7 RNA polymerase (Fig 2A and 2B). For ultrastructural analysis we used chemical fixation (CF) or high-pressure freezing (HPF) (Fig 2C). Regardless of the fixation technique, we identified basically the same types of membrane alterations observed in phenotype 1 of MNV infected cells: vesiculated areas with SMVs, DMVs and MMVs, again in close proximity to LDs, whereas phenotype 2 (complex membrane structures lacking organized morphology) was not found upon expression of ORF1.

We concluded that expression of ORF1 generates membrane alterations comparable to replication organelles found in MNV infection. Therefore, ORF1 expression seemed a valid model to study the morphology of huNoV induced membrane alterations.

### Establishment of an ORF1 polyprotein expression model of three GII.4 outbreak strains of HuNoV

We used ORF1 sequences of three GII.4 strains associated with pandemic outbreaks: a Den Haag 2006b variant (DH) [49], a New Orleans 2009 variant (NO) [50] and a Sydney 2012 variant (Syd) [51]. We first tested the expression of the different ORF1 proteins and their processing to assess the integrity of the polyproteins. In a coupled *in vitro* transcription/translation system (S3A Fig) most of NS-proteins remained buried in precursors, which according to their sizes could represent the entire ORF1 and NS4-NS7 (S3A Fig). Mature cleavage products were only found for NS1-2 and/or NS3, however the size of these proteins was almost identical. This result was in line with previous data studying ORF1 *in vitro* processing of GII.4 [43,52]. In addition, we assessed polyprotein expression and processing by Western blotting (WB) after transfection of the plasmids encoding the three ORF1 proteins into Huh7 T7 cells. We could detect cleavage products corresponding to NS3, (S3B Fig), NS7 (S3C Fig) and NS1-2, the latter by expressing N-terminal eGFP tagged ORF1 from the NO isolate since we lacked a specific antibody (S3G Fig). No distinct cleavage products were observed for NS4, NS5 and NS6 (S3D–S3F Fig), indicating that they might be retained in relatively stable precursor proteins, as suggested by *in vitro* translation.

**Fig 3:**
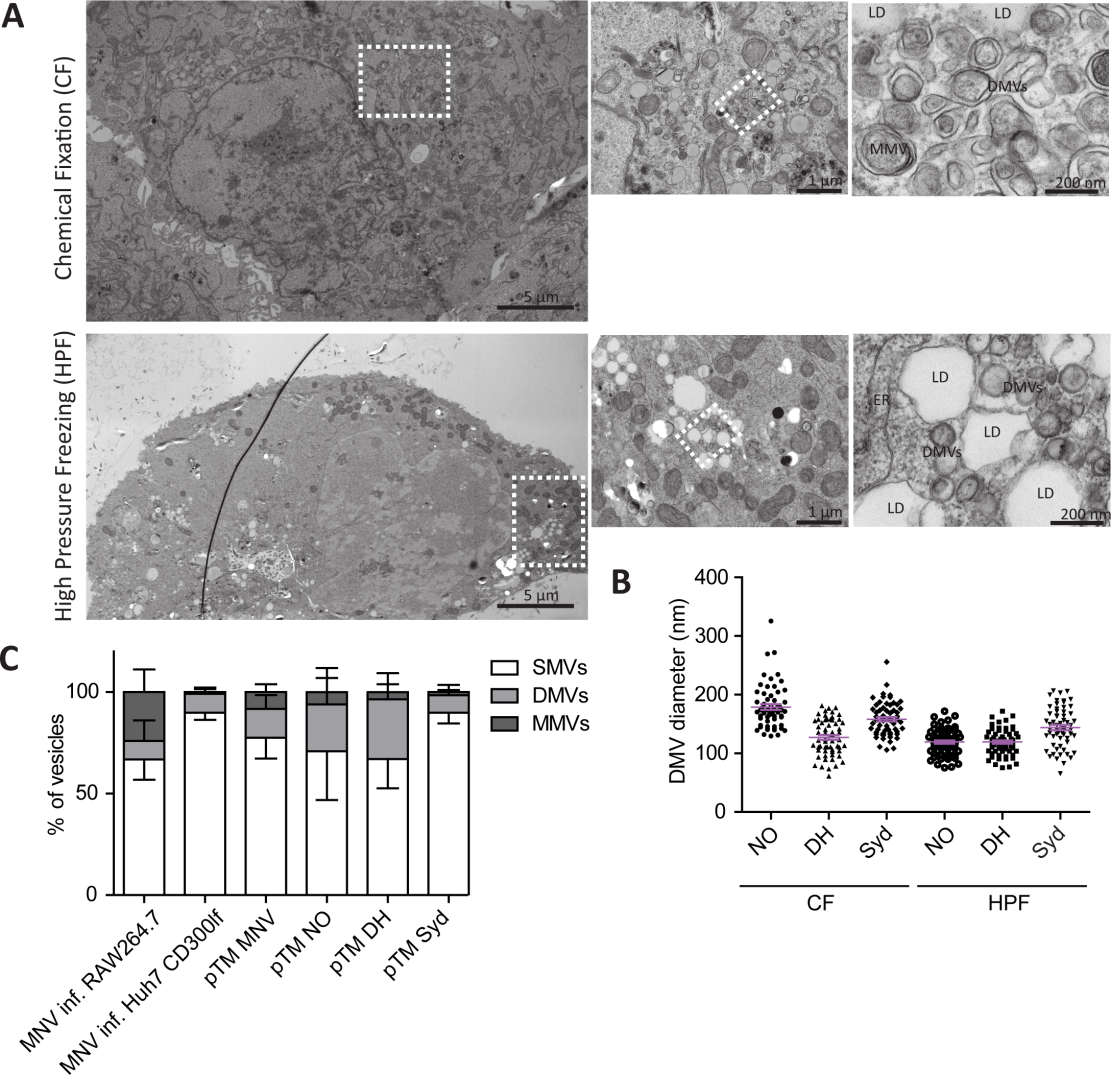
Ultrastructural analysis of pTM ORF1 expression. (A) Huh7 T7 cells transfected with a construct expressing NO ORF1 were processed for analysis by transmission EM 20 h p.t., using HPF and CF, as indicated (for further detail see the legend to Fig 2C). Double membrane vesicles (DMVs), multi membrane vesicles (MMVs) and lipid droplets (LDs) are indicated. (B) Comparison of DMV sizes induced by ORF1 expression of the New Orleans (NO), Den Haag (DH) and Sydney (Syd) strains, using both fixation protocols. The diameter of 60 DMVs for each condition was determined. Mean values and SEM are shown in violet. (C) Relative abundance of SMVs, DMVs and MMVs in cells expressing ORF1 of the different GII.4 isolates or MNV and in MNV infected cells after chemical fixation. ORF1 expressing cells and MNV infected cells were identified by the appearance of typical virus induced membrane structures and all vesicles in the respective cell sections were classified and counted. Mean values and SD from at least six cells per condition. For clarity, error bars are shown below the column for SMVs and above the column for DMVs and MMVs.

To investigate membrane alterations resulting from huNoV nonstructural proteins, we expressed the complete ORF1 protein of the three GII.4 isolates in Huh7 T7 cells and performed EM analysis (Fig 3, S4 Fig). As for MNV, the expression of ORF1 polyproteins resulted in the formation of complex vesicular structures for all three GII.4 isolates (Fig 3, S4 Fig), similar to those found in MNV-infected cells (Fig 1, S2 Fig) and irrespective of the fixation technique used. The average diameter of DMVs was approximately 100–200 nm (Fig 3B), and resembled structures found upon HCV and picornavirus infection [28,31,53–56]. Most membrane alterations induced by ORF1 expression in Huh7 cells were again found in close association with LDs, similar to MNV. Overall, no consistent differences were found between ORF1 expression and infection regarding the ratio of SMVs, DMVs and MMVs (Fig 3C). However, high variability in vesicles size was observed among cells of the same condition, likely due to differences in protein abundance, time of infection, cell type, etc. Still, SMVs were by far the most abundant vesicle species in all conditions.

In summary, expression of ORF1 of different GII.4 isolates gave rise to a complex set of membrane alterations independent of RNA replication, but similar to structures found in MNV-infected cells. Altogether our data suggested that norovirus replication organelles might belong to the DMV-morphotype, comparable to those observed for enteroviruses and HCV.

### Electron Tomography (ET) of cells expressing GII.4 NO ORF1

We focused our subsequent analyses on one of the three GII.4 strains (NO), since neither polyprotein processing nor the ultrastructural analysis revealed distinct differences upon the expression of ORF1 among the three strains.

**Fig 4:**
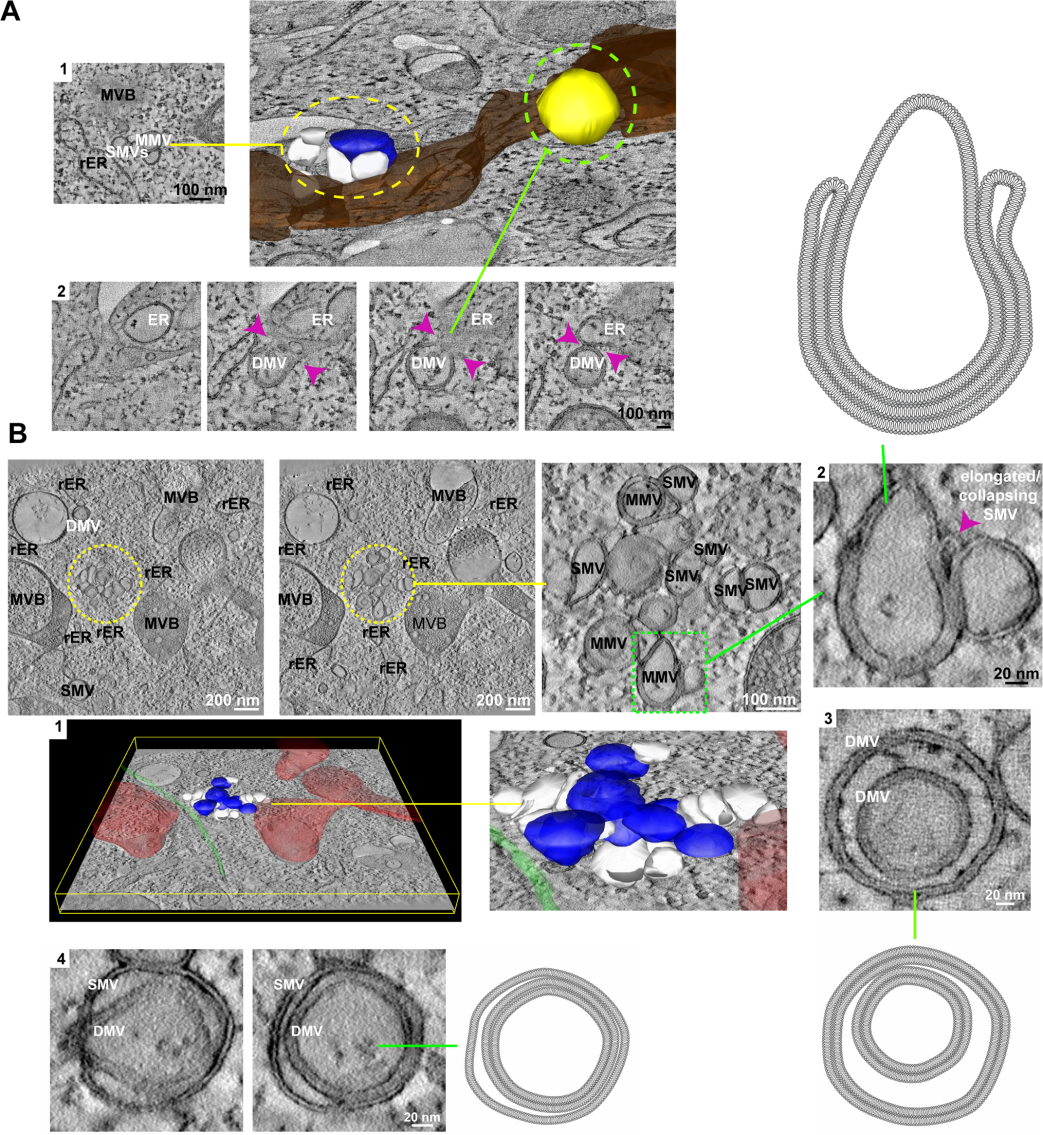
Electron tomography of vesicle clusters induced by ORF1 of the NO strain. (A) 3D rendering of a dual-axis tomogram of S1 Movie showing ER cisternae (in dark brown), a cluster of single (in white) and multi membrane (in blue) vesicles, and a double membrane vesicle (in yellow). A tomographic *xy* slice of the yellow dashed area containing the cluster of vesicles is shown in (1). Several virtual slices extracted from the tomogram of the green dashed area are shown in (2). Note the continuity of the DMV membranes with the ER membrane (pink arrowheads). (B) Serial single *xy* slices through the tomogram shown in S2 Movie revealing the different membrane numbers of the vesicles within the cluster: from one lipid bilayer (SMVs) to two (DMVs) or more than two (MMVs). (1) Left: 3D rendering of the same tomogram showing a cluster of single (in white) and multi-membrane (in blue) vesicles in close proximity to late endosomes (in red) and a microtubule (in green). Right: Higher magnification picture of the cluster of vesicles. (2) MMVs are composed of several single membrane tubules that close up around a single membrane vesicle. rER, rough endoplasmic reticulum; SMV, single membrane vesicle; DMV, double membrane vesicle; MMV, multi membrane vesicle; MVB, multivesicular body. Note that we defined endosomes or MVBs as rounded organelles delimited from the cytosol by one lipid bilayer, containing a highly heterogeneous lumen composed of multiple vesicles with different sizes and electron densities. ALSs were defined as rounded organelles having two lipid bilayers that separate them from the cytosol and a diameter larger than 300 nm. Their lumen, in contrast to MVBs, was only composed of cytosolic content and/or one engulfed vesicle. We cannot rule out, however, that ALSs are larger DMVs.

To gain deeper insights into the morphology and biogenesis of ORF1-induced membrane structures we further analyzed tomograms of cells expressing ORF1 of the NO strain fixed by high pressure freezing (S1–S5 Movies, Fig 4A and 4B and S5 Fig), allowing a better preservation of the cell membranes. Areas appearing as simple accumulations of SMVs, DMVs and MMVs revealed complex structures in close proximity to ER sheets, including clusters of interwoven vesicles delimited by one or several lipid bilayers (S1 and S2 Movies and Fig 4A and 4B). In addition, we found double membrane vesicles (DMVs) connecting to multivesicular bodies (MVBs) or late endosomes (S1 and S3 Movies and S5A and S5B Fig) and autophagosome-like structures (ALS, S1 and S3 Movies and S5C Fig).

Since clusters of SMVs, DMVs and MMVs most closely resembled the organization of the MNV replication sites in infected cells, we rendered these areas to address their 3D organization (Fig 4A and 4B, S4 and S5 Movies). SMVs (white), DMVs (yellow) and MMVs (blue) all appeared rather vesicular than tubular, were tightly attached to each other and mostly found adjacent to ER cisternae. In some serial slices, the membrane of a DMV was found still in continuation with the ER (Fig 4A, panel 2). MMVs were mainly generated by enwrapping of SMVs with tubular structures, most likely elongated or collapsed SMVs, appearing as multilamellar vesicles in cross-sections (Fig 4B, panel 2). Alternatively MMVs were originated as SMVs or DMVs engulfing pre-existing DMVs (Fig 4B, panel 3, 4). Overall, the morphology and complexity of the membrane alterations, as well as their biogenesis, appeared very reminiscent of later stages of the enterovirus replication sites [53–56].

Altogether, the ET analysis revealed complex interwoven vesicular structures adjacent to the ER, with one or several membrane bilayers, appearing as SMVs, DMVs and MMVs. MMVs were likely generated by enwrapping of SMVs with elongated SMVs, very similar to the mechanism proposed for enteroviruses.

### The N-terminal protein GII.4 NS1-2 localizes at the ER and induces tubular ER protrusions

Little is known so far about the contribution of individual norovirus NS proteins to the biogenesis of the viral replication complex. We therefore fused NS1-2, NS3 and NS4, known to be associated with membranes [13], N-terminally with eGFP, to study their propensity to induce membrane alterations by correlative light and electron microscopy (CLEM). We first confirmed by WB the expression of stable fusion proteins and the absence of free eGFP (S6A and S6B Fig).

**Fig 5:**
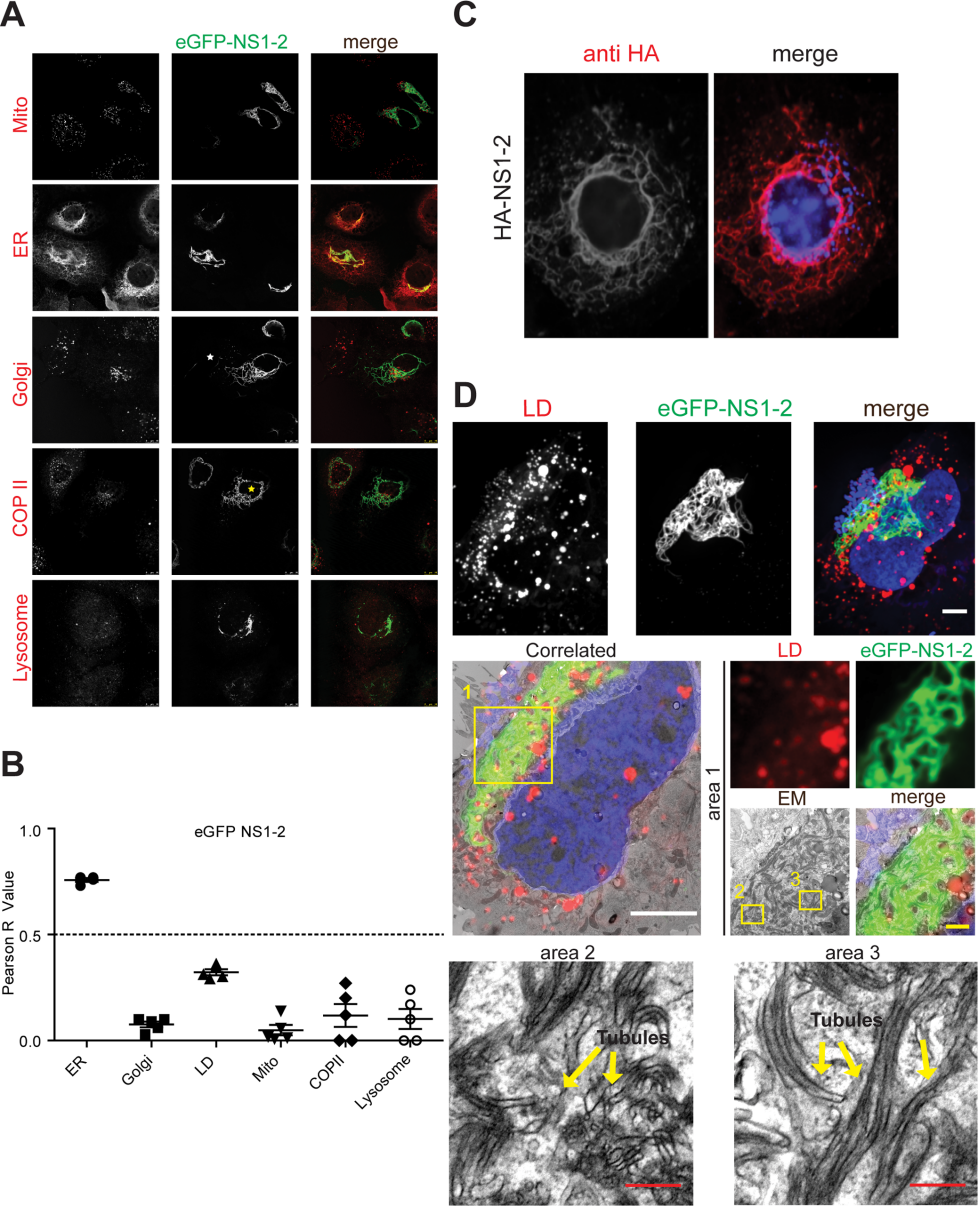
Subcellular localization of NS1-2 analyzed by IF and CLEM. A plasmid encoding eGFP-NS1-2 (green) was transfected into Huh7-T7 cells. Twenty hours post transfection cells were fixed and analyzed by confocal microscopy (A, B) or CLEM (D). Cellular markers (red) were stained using monoclonal or polyclonal antibodies, LDs were stained with LipidTox and Mitochondria were stained with Mitotracker. A white and a yellow asterisk mark a cell with a focal and an intermediate focal/filamentous distribution of NS1-2, respectively. Mito, mitochondria. (B) Pearson correlation of the eGFP-NS1-2 signal with different cellular markers shown in (A). Each dot represents a single cell. (C) HA-NS1-2 was expressed in Huh7-T7 cells and detected by immunofluorescence using a HA-specific antibody (red). Nuclei were counterstained with DAPI (right panel, blue). (D) For CLEM, cells were seeded onto gridded coverslips, fixed and subjected to optical sectioning using a confocal microscope. Maximum-intensity Z-projection of a selected cell is shown on the top. eGFP-NS1-2 signal is depicted in green, LDs in red and the nucleus (DAPI) in blue. Samples were subsequently processed for electron microscopy by using the coordinates etched onto the surface of the gridded coverslips to record the position of the selected cells. The correlated panel was obtained by re-orientation and superimposition of light and electron micrographs as described in M&M. Boxed and numbered areas are shown in higher magnification in subsequent panels. White scale bars, 5 μm. Yellow scale bar, 1 μm. Red scale bars, 200 nm.

The N-terminal protein of norovirus ORF1 is thought to induce membrane rearrangements and is considered to be involved in replication complex formation (reviewed in [8]). First, we examined the localization of eGFP-NS1-2 with respect to subcellular markers by immunofluorescence (Fig 5A). Interestingly, eGFP-NS1-2 had a very peculiar filamentous subcellular distribution in most cells (Fig 5A). A minority of cells showed a focal distribution of NS1-2 or an intermediate phenotype (Fig 5A, white and yellow asterisk, respectively). Since the filamentous localization was observed for N-terminally HA-tagged NS1-2, an artifact caused by eGFP fusion can be excluded (Fig 5C). We found a significant co-localization of NS1-2 with a marker of the ER, judged by Pearson correlation values above 0.5 (Fig 5A and 5B). Next, we used CLEM to determine the ultrastructural morphology of NS1-2-GFP positive structures (Fig 5D). Here, regions with strong eGFP fluorescence, indicating high NS1-2 expression, were represented by a network of tubular membrane protrusions. The absence of ribosomes associated with these structures, and the general co-localization of NS1-2 with an ER marker indicated that these membrane protrusions originated from the smooth ER. It is interesting to note that similar tubular structures can be induced by overexpression of ER-shaping proteins such as REEP1 and CLIMP-63 through direct interaction with microtubules [57]. However, we found no indication for a co-localization of NS1-2 induced ER-tubules with microtubules or intermediate filaments (S6C and S6D Fig). In addition we analyzed co-localization of eGFP-NS1-2 and NS3 upon expression of eGFP-ORF1 (S6E and S6F Fig) to assess the impact of the polyprotein on eGFP-NS1-2 localization. Interestingly, we found a variety of phenotypes in various cells, ranging from a focal distribution of both proteins (upper panel) to the filamentous localization of NS1-2 observed upon individual expression (lowest panel). This result indicated a mutual impact of the NS proteins on their subcellular localization, retaining significant co-localization in all cases (S6F Fig), as reported for MNV [15]. Since the distribution of GII.4 NS1-2 was very different from the pattern reported for NS1/2 of MNV [13], we further analyzed eGFP-NS1/2 of MNV by CLEM (S7 Fig). Interestingly and in concordance with literature, NS1/2 of MNV was widely distributed throughout the cell and co-localized with the ER (S7B Fig). However, we found no indications for specific membrane structures induced by MNV NS1/2 (S7C Fig) comparable to those found for GII.4 NO. The MNV NS1/2 signal could be often correlated with membranes surrounding LDs (S7C Fig), which may represent the ring like structures described in a previous study [13].

Taken together, eGFP-NS1-2 of GII.4 induced tubular protrusions of membranes likely derived from smooth ER in a focused, mainly perinuclear area. This is in contrast to MNV NS1/2, which is widely distributed on the ER. This suggests that NS1-2 of GII.4 probably does not directly induce the vesicular membrane rearrangements observed upon ORF1 expression, but rather may contribute to the proliferation of membranes engaged in replication complex formation. Our results further illustrate differences in the subcellular localization and possible functions of NS1-2 proteins from different norovirus genogroups.

### GII.4 NS3 is associated with membranes surrounding LD membranes, the Golgi and rough ER

**Fig 6:**
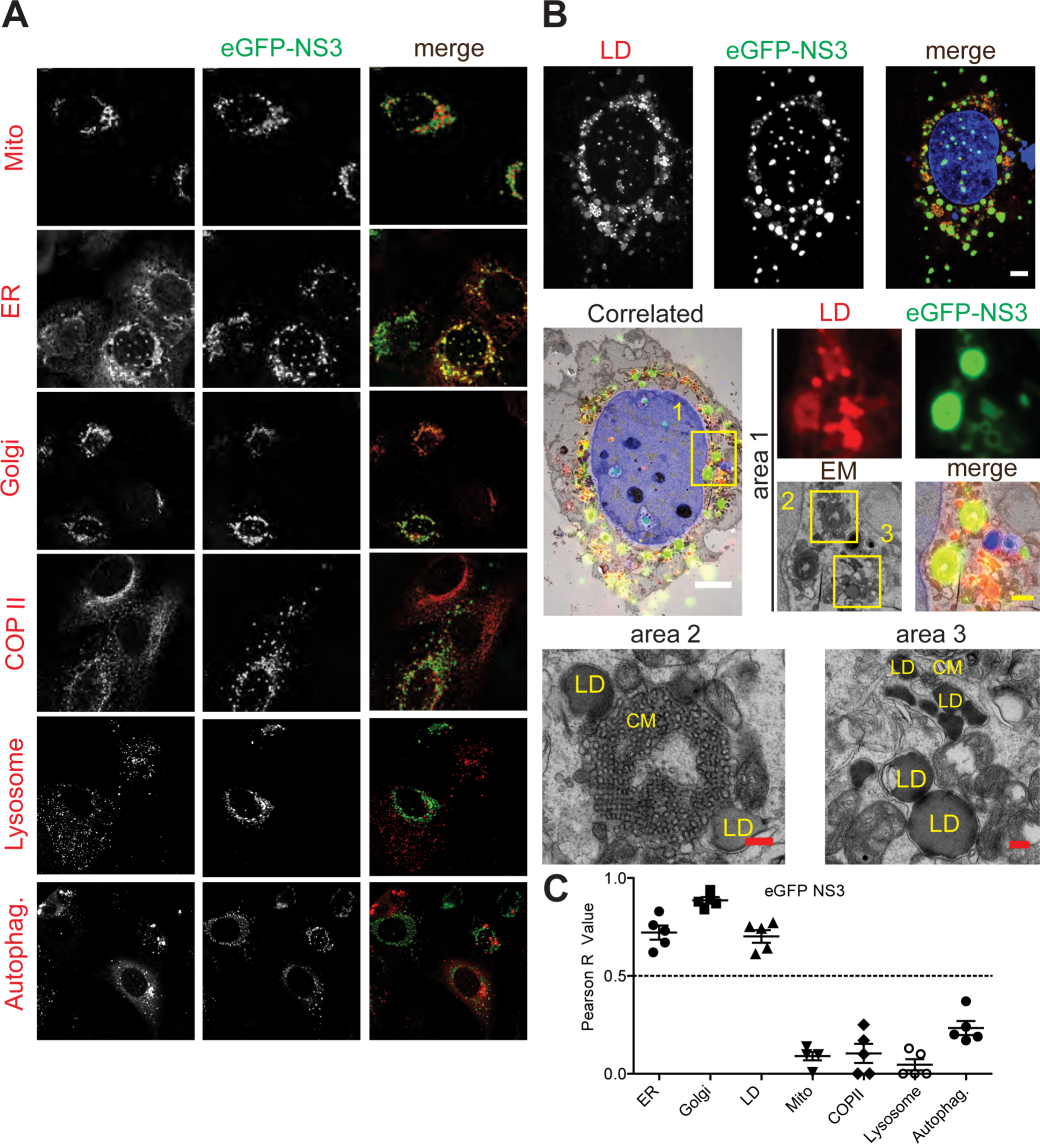
Subcellular localization of eGFP-NS3 analyzed by IF and CLEM. A plasmid encoding eGFP-NS3 (green) was transfected into Huh7-T7 cells. Twenty hours post transfection, cells were fixed and analyzed by confocal microscopy (A) or CLEM (B). (A) Cellular markers are depicted in red and eGFP-NS3 in green in the merge panels. Mito, mitochondria; Autophag., autophagosome. (B) Huh7-T7 cells expressing eGFP-NS3 were processed for CLEM. For further details see the legend to Fig 5D and M&M. Maximum-intensity Z-projection of selected cell is shown on the top. The eGFP-NS3 signal is depicted in green, lipid droplets (LD) in red and the nucleus (DAPI) in blue. Boxed and numbered areas are shown in higher magnification in subsequent panels. CM, Convoluted Membranes. White scale bars, 5 μm. Yellow scale bar, 1 μm. Red scale bars, 200 nm. (C) Pearson correlation of the eGFP-NS3 signal with different cellular markers shown in (A). Each dot represents a single cell.

We next analyzed the co-localization of eGFP-NS3 of GII.4 NO with different markers of subcellular compartments (Fig 6A). We observed a distinct, rather dot-like staining pattern for NS3 (Fig 6A and 6B), which was similar to the pattern described for MNV NS3 [13]. NS3 significantly co-localized with markers of the Golgi apparatus, rough ER and LDs (Fig 6A and 6C). We also characterized the subcellular localization of NS3 upon expression of ORF1 (S8A–S8C Fig). Again we found some co-localization of NS3 with ER and LDs (S8A and S8C Fig), albeit to a lesser extent as in case of individually expressed NS3. In contrast, very little co-localization with several other markers of membranous intracellular organelles was observed, including Golgi apparatus (S8C Fig), suggesting again a mutual impact of the NS proteins on their subcellular localization. The tight association of GII.4 NS3 with LDs was also validated in CLEM experiments (Fig 6B). Donut like structures of NS3 observed in IF were indeed NS3-studded membrane layers surrounding LDs (area 1). Interestingly, large foci with strong NS3 but weak LD signal were found to represent highly ordered membrane proliferations and were often observed in close proximity to one or more LDs (area 2, 3). Such convoluted membranes were similar to previously described OSER (organized smooth ER) membranes with cubic symmetry (reviewed in [58,59]). However, we did not observe these structures upon ORF1 expression (Fig 3). Overall, our findings indicate that GII.4 NS3 was found on different membrane compartments of the secretory pathway and was closely associated to intracellular lipid storage compartments.

### GII.4 NS4 is capable of inducing the formation of DMVs as well as other types of membrane alterations

**Fig 7:**
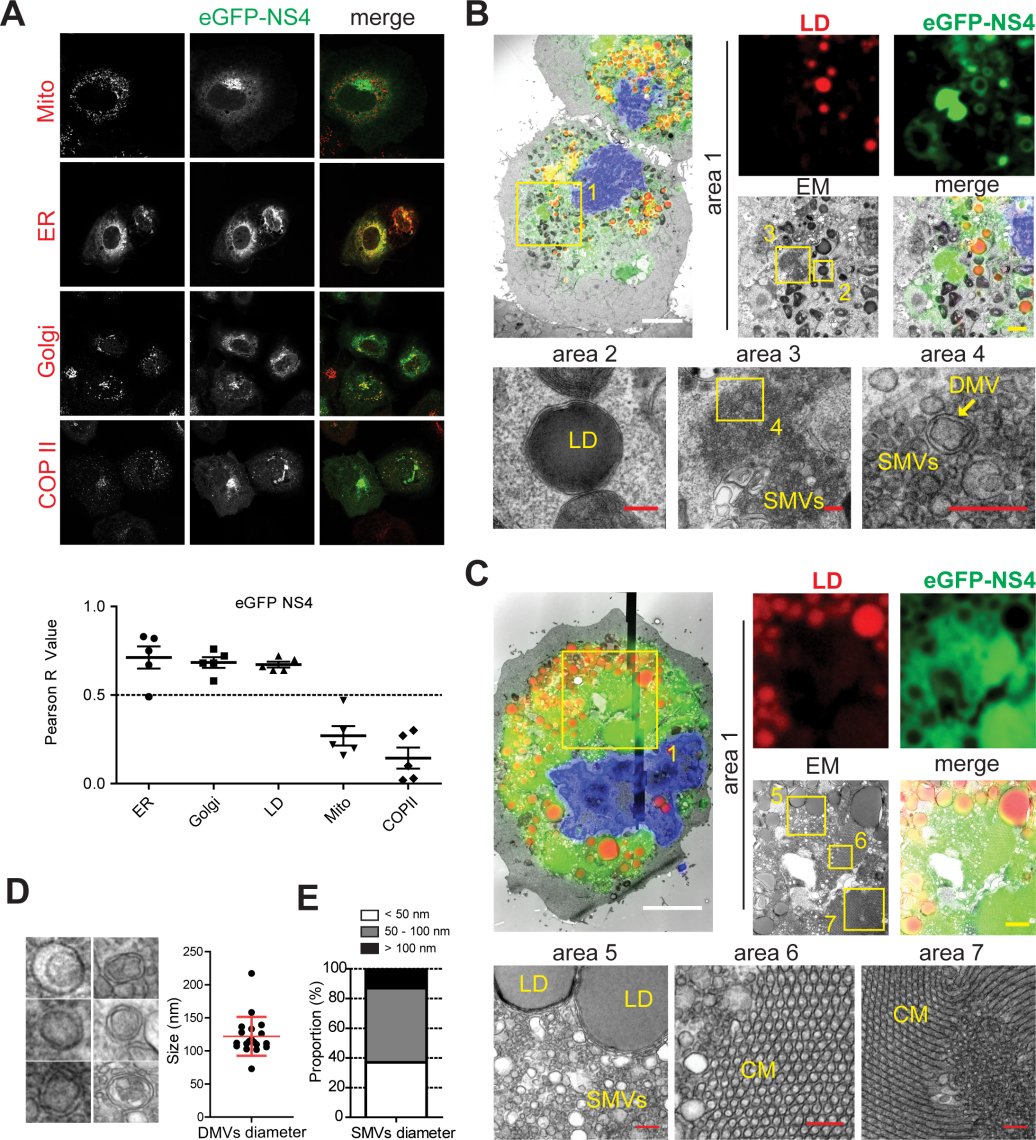
Subcellular localization of eGFP-NS4 analyzed by IF and CLEM. A plasmid encoding eGFP-NS4 (green) was transfected into Huh7-T7 cells. Twenty hours post transfection cells were fixed and analyzed by confocal microscopy (A) or CLEM (B-C). (A) Cellular markers are shown in red and eGFP-NS4 in green in the merge panels. Pearson correlation of the eGFP-NS4 signal with different cellular markers is given below, each dot representing a single cell. Mito, mitochondria. (B-C) Huh7-T7 cells expressing eGFP-NS4 were processed for CLEM. For further details see the legend to Fig 5D and M&M. eGFP-NS4 signal is depicted in green, lipid droplets (LD) in red and the nucleus (DAPI) in blue. Boxed and numbered areas are shown in higher magnification in subsequent panels. CM, convoluted membranes; SMV, single membrane vesicle; DMV, double membrane vesicle. White scale bars, 5 μm. yellow scale bar, 1 μm. red scale bars, 200 nm. (D) Higher magnification images showing eGFP-NS4 induced DMVs (left panel). Quantification of the DMVs diameters calculated from 19 DMVs from three different cells. Mean and SD are shown. (E) Diameter of SMVs generated by eGFP-NS4 expression. SMVs were grouped into three different size classes as indicated and their relative proportion is given. Data are based on 350 individual, randomly chosen SMVs in three different cells.

The fluorescence pattern of eGFP-NS3 and -NS4 was quite similar, revealing a dot like pattern with donut like and filled structures (Fig 7A) located mainly in the perinuclear area. Still, the eGFP-NS4 signal tended to accumulate in larger clusters compared to the majority of eGFP-NS3 (Fig 7A and 7B), but also co-localized with markers of ER, Golgi apparatus and LDs (Fig 7A and 7B). CLEM analysis identified several interesting types of membrane alterations in areas with strong eGFP signal, in agreement with the idea that NS4 is a key driver in the formation of the norovirus replication compartment. Donut like structures were found to be membranes tightly associated with LDs (Fig 7B, area 2), similar to those found for NS3 (Fig 6B). In addition, large eGFP-NS4 positive foci found in close proximity to LDs consisted of vesicle clusters composed of DMVs and SMVs (Fig 7B, area 3, 4; 7C, area 5). The size and morphology of NS4 induced DMVs was very similar to those observed upon expression of the polyprotein (Fig 7D compared to Fig 3B), but their abundance was apparently lower. In contrast, SMVs were much more abundant and heterogeneous in size, with a diameter ranging from 50 nm to 300 nm, although more than ~80% had a diameter <100 nm (Fig 7E). These data suggested that NS4 on its own was capable of inducing vesicle accumulations reminiscent of vesicle clusters of the GII.4 replication compartment. Finally, similarly to NS3, highly fluorescent clusters of NS4 within the cells corresponded to regions forming regularly shaped membrane lattices (Fig 7C, areas 6 and 7). However, NS4 expression resulted in membranes aligned predominantly in tubules with hexagonal symmetry (Fig 7C, area 6). This hexagonal symmetry appeared very similar to the arrangement observed upon overexpression of the hydroxy-methylglutaryl (HMG)-CoA reductase [60], although regions with cubic symmetry were also observed (Fig 7C, area 7). Furthermore, NS4 induced crystalline membrane structures were found in proximity to vesicle clusters (Fig 7C, area 1), suggesting that their formation might be concentration dependent and a consequence of very high local concentrations of NS4.

In essence, our results indicated that NS4 was a key factor in the biogenesis of GII.4 induced membrane alterations. Specifically, the sole expression of NS4 was sufficient to induce several different types of membrane structures, including SMVs, DMVs, as well as geometric membrane lattices not found in infected cells.

### Structural predictions of GII.4 NS proteins NS1-2, NS3 and NS4 and functional analysis of point mutations in the active center of the putative hydrolase domain of NS1-2

**Fig 8:**
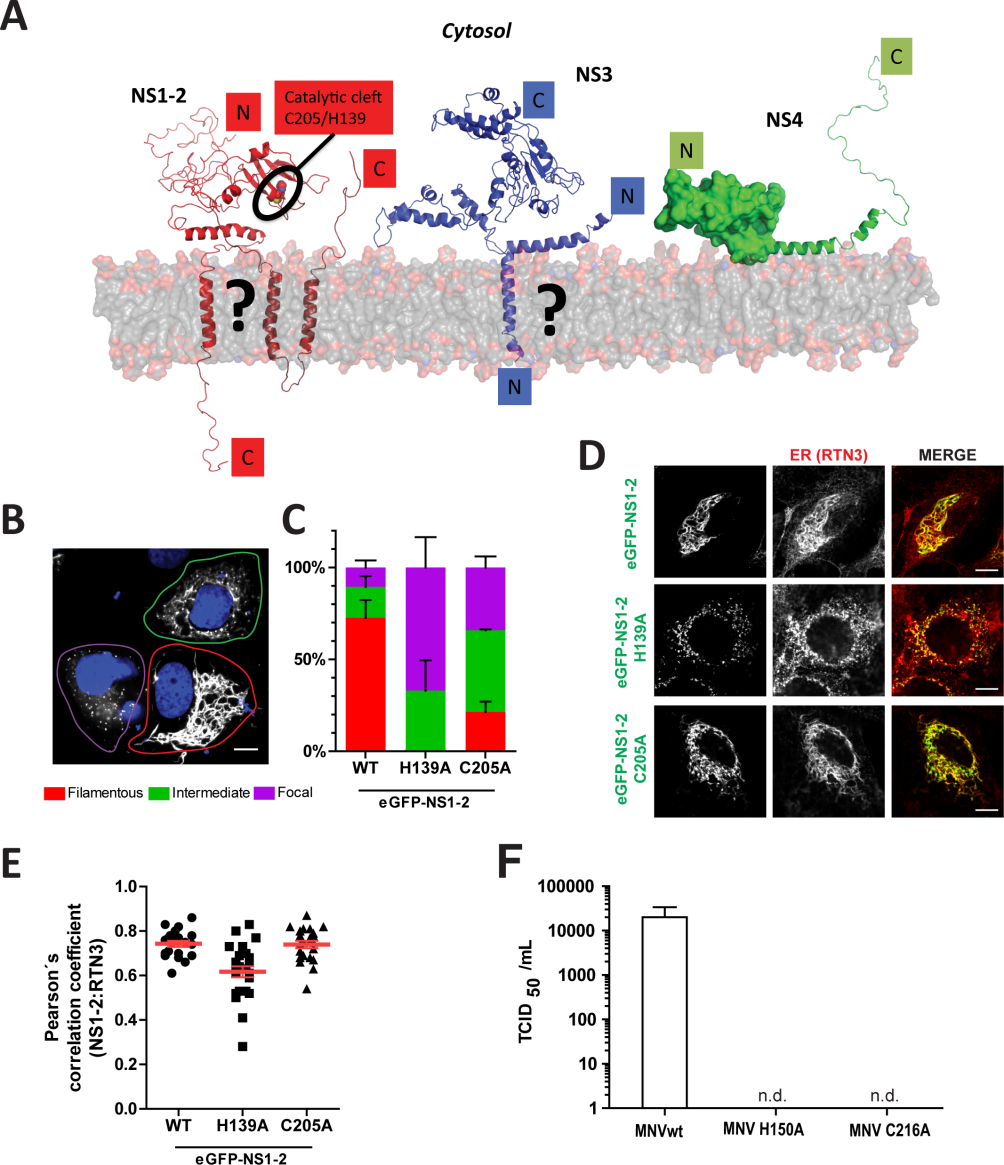
Modeling of GII.4 NS1-2, NS3 and NS4 and analysis of mutants of the putative hydrolase domain. (A) Modeling. Parts for which modeling is most reliable are displayed in secondary structure representation: First the two domains that were homology modeled, namely the central domain of NS1-2 (the catalytic C205 and H139 residues are displayed as spheres) and the NTPase domain of NS3; Second the membrane association helices of the three proteins. For NS1-2, the alternate possibilities for its C-terminal membrane association, as one membrane-peripheral and one transmembrane helix, or as two transmembrane helices, are indicated by a question mark. Similarly for NS3, the alternate possibilities for its N-terminal helix as transmembrane or as membrane-peripheral are indicated with a question mark. The natively unfolded N-terminus of NS1-2 and C-terminus of NS4 are displayed as random coils. The N-terminus of NS4, folded according to secondary structure predictions but with no homolog of known structure, is displayed as a blob with a putative amphipathic helix leading to the unstructured C-terminus in secondary structure representation. (B-E) pTM plasmids encoding NS1-2 variants as indicated N-terminally fused to eGFP were transfected into Huh7-T7 cells and harvested 20 hours post transfection. Cells were fixed and permeabilized with Triton-X100 (0.5%), and ER (red) was stained using a RTN-3 monoclonal antibody. (B) Cells expressing eGFP NS1-2 C205A, with representative examples of the filamentous, intermediate and focal phenotype, encircled in red, green and violet, respectively. (C) Percentage of cells showing a filamentous, focal or intermediate phenotype for cells expressing eGFP-NS1-2 wt or the indicated mutant. Mean values and SD from at least a total of 100 cells from two independent experiments are shown for each condition. (D) A representative picture showing the filamentous phenotype for wt, the focal phenotype for mutant H139A and the intermediate phenotype for mutant C205A. (E) Pearson correlation of the eGFP signal with ER marker shown in (D); each dot representing one cell. (F) TCID50/ml values obtained from wt and mutant MNV variants in HEK293-T cells. Plasmids encoding a wildtype or mutant MNV full-length cDNA clone were transfected into HEK293T cells. Virions were harvested 72 hours post transfection and titrated on RAW 264.7 cells. Titers of infectious virus was determined by TCID50 assay and calculated using the Spearman-Karber method. Values represent the mean of three independent experiments with error bars representing the standard deviation. n.d.: not detectable. Note that positions H139 and C205 in GII.4 NS1-2 correspond to H150 and C216 in the MNV genome.

Our results obtained from individual expression of eGFP-NS1-2, NS3 and NS4 of GII.4 strain NO revealed that they were indeed associated with membranes. Since the functions of all three proteins, in particular NS1-2 and NS4, are widely enigmatic, we next aimed to generate structural models based on secondary structure analysis and homology searches, allowing for the development of hypotheses accessible to experimental validation. There are no close homologs of known structures for these three proteins, but advanced search methods unambiguously detect distant homologs (20% sequence identity) for parts of both NS1-2 and NS3. These together with secondary structure predictions show that the 334-residue NS1-2 can be described as a three-partite protein with an unstructured N-terminus (residues 1–110) followed by a papain-like thiol hydrolase domain (residues *ca* 120–230) that is related to a family of phospholipases and acyltransferases [61], and finally a hydrophobic domain (residues *ca* 250–310) with one or possibly two transmembrane helices. We can thus draw two possible topologies for membrane association of NS1-2 (Fig 8A). Interestingly, the catalytic cysteine and histidine of the putative thiol hydrolase are both present in NS1-2 as C205 and H139. The 366-residue NS3 is a distant homolog of AAA ATPases with an extra 50 residues at the N-terminus comprising a hydrophobic helix that could be transmembrane. Again, we have two possibilities for NS3 membrane association. Finally, we could find no homolog of known structure for the 179-residue NS4, but secondary structure predictions show that its approximately 140 N-terminal residues are highly structured and end in an amphipathic helix connecting to a natively unfolded C-terminus (Fig 8A).

Based on these predictions we finally aimed to gain some evidence for the putative hydrolase domain and its importance for membrane protrusions induced by NS1-2 of GII.4. To this end we generated mutants of the highly conserved proposed catalytic residues H139 and C205 in the context of eGFP-NS1-2. Since these residues are invariant in all norovirus genogroups, we used MNV as a surrogate model to study their functional importance for norovirus replication. Interestingly, both NS1-2 mutants affected the abundance of the filamentous phenotype (Fig 8B–8D), arguing for a contribution of the putative hydrolase domain to the generation of tubular ER protrusions. Albeit mutations H139A and C205A both resulted in an increased abundance of the intermediate phenotype, or a focal distribution of eGFP-NS1-2, all variants still remained localized to the ER (Fig 8E). Importantly, both residues were indeed essential for norovirus replication, as mutations at the corresponding positions H150A and C216A of MNV NS1-2 abrogated the production of infectious virus (Fig 8F).

In summary, our analysis of individually expressed NS1-2, NS3 and NS4 suggest that these proteins, in particular NS4, were the main drivers of replication complex formation for GII.4. The structural models proposing their membrane topology will allow more in depth studies of their precise functions. Our data further suggest a role of the predicted hydrolase domain in the membrane shaping activity of NS1-2, but not to the general localization to the ER.

## Discussion

In this study, we analyzed membrane alterations induced by ORF1 expression of clinically relevant GII.4 isolates and by the individually expressed NS-proteins. ORF1 expression generated vesicle accumulations comparable to those observed in MNV infected cells, mainly consisting of SMVs, DMVs and MMVs. Therefore, norovirus-induced membrane alterations can be generated in the absence of active RNA synthesis, and are reminiscent of picornaviruses and HCV [28,31,53–56]. Our data indicate that NS1-2, NS3 and NS4 are the main drivers in the formation of GII.4 replication organelles. However, only NS4 generated SMVs and DMVs similar to those observed upon ORF1 expression and MNV infection. These data provide the first experimental evidence for the hypothesis that NS4 might be the key factor in the morphogenesis of norovirus replication organelles (reviewed in [8]).

Previous studies on the ultrastructure of MNV replication organelles in RAW 264.7 cells reported on the appearance of vesiculated areas consisting of SMVs and DMVs at 12h post infection, progressing to a complete destruction of ER and Golgi at later time points, coinciding with the accumulation of virions [44]. We found in principle the same two phenotypes upon MNV infection of Huh7-CD300lf cells and in RAW 264.7 cells. ORF1 expression induced structures very similar to the phenotype lacking virions, likely corresponding to an early/intermediate infection stage, but did not progress into the complex endomembrane system observed concomitantly to intracellular virions appearance. Progression to the late stages found in infection might require the presence of the structural proteins or resemble cytopathic effects induced by infection, but not by ORF1 expression. Interestingly, we found no obvious difference in the membrane rearrangements induced by ORF1 or MNV compared to any of the three GII.4 isolates included in this study, arguing for similar mechanisms driving these processes in various norovirus genogroups and validating MNV as a useful surrogate model to study particular aspects of general norovirus biology. Still, our data obtained upon GII.4 ORF1 expression will ultimately require validation in a cell culture model with *bona fide* RNA replication, such as the recently established enteric organoid cultures [43] or infection of B cells [42]. The same holds true for the functional significance of the LD association we found for GII.4 NS3 and NS4. While LDs are clearly not essential for MNV replication, since they are not detectable in RAW 264.7 cells, we found them close to all virus induced membrane alterations in Huh7 cells, both, upon expression of ORF1 and upon MNV infection. Whether this is co-incidence due to high LD abundance in Huh7 cells or whether LDs have a functional significance in GII.4 replication remains to be determined. At this point we have not been able to detect membrane alterations that could unequivocally be assigned to norovirus infection by EM in norovirus infected enteroid cultures (S. Boulant, personal communication), likely due to the yet limited efficiency of this model and the high percentage of infected cells required for a thorough EM analysis. However, further optimization of culture conditions will hopefully allow such studies in the future, as well as the establishment of a reverse genetics model for GII.4 isolates.

Our ultrastructural analysis does not allow drawing firm conclusions on the origin and biogenesis of GII.4 induced SMVs, since we did not observe such vesicles directly connected to cellular membranes. In contrast to PV, we could not find evidence for an interconnected tubular network [55]. Most of our evidence points to membranes of the ER as the origin of SMVs, similar to MNV [15], due to the co-localization of NS1-2 and NS3 with ER membranes when expressed in the context of ORF1. Furthermore, our tomograms reveal a close proximity of the induced vesicles to ER membranes. Finally, individually expressed NS1-2, NS3 and NS4 all at least in part co-localize to and rearrange ER membranes. Therefore, huNoV, like many other viruses, may hijack this cell organelle to generate its replication organelles (reviewed in [62]). Regarding DMVs and MMVs, we were able to identify intermediate structures. Many vesicles appearing as DMVs in a single plane were in fact MMVs *in stato nascendi*, generated by enwrapping vesicles with collapsed, elongated SMVs or ER cisternae. DMVs might originate as well from collapsing SMVs. This indeed closely resembles the mechanisms demonstrated for picornaviruses [55,56]. Finally, our tomograms also showed a variety of complex structures (e.g. MVBs, ALS) that might be linked to the biogenesis of these vesicles, however these structures are currently difficult to interpret regarding their functional significance. Overall, our data suggest that norovirus induced membrane alterations are very closely related to picornavirus replication organelles with respect to both morphology and biogenesis. The relatively low abundance of DMVs and MMVs further argues for a non-essential role in norovirus infection, again in line with reports on picornaviruses, where replication most likely occurs on single membrane structures, whereas the appearance of DMVs is associated with later stages of infection [55,56].

Expression of the individual nonstructural proteins fused to eGFP and subsequent analysis by CLEM revealed striking phenotypes for NS1-2, NS3 and NS4, which are very poorly understood regarding specific functions in viral RNA replication and topology (Fig 8A). The sequence of NS1-2 is highly divergent between different norovirus genogroups with no specific function assigned thus far. Bioinformatic analysis proposed an unstructured N-terminal region, which was confirmed biochemically [9], a central domain with potential hydrolase function [10] and a C-terminal transmembrane domain [9,63]. Norwalk virus NS1-2 was shown to disrupt the Golgi apparatus [11], whereas MNV NS1-2 mainly localizes to the ER [13]. Our data now show a very peculiar filamentous localization of GII.4 NS1-2 which could be identified as tubular protrusions of the smooth ER when analyzed by CLEM. Similar structures have been observed upon overexpression of cellular ER remodeling proteins such as REEP1 and CLIMP-63 through direct interaction with microtubules [57], which appear to be the driving force in protruding the tubules. In the case of NS1-2, we found no co-localization with microtubules or intermediate filaments, therefore it is currently not clear how the ER-tubules are expanded. Since mutations in the active site of the putative hydrolase domain severely affected the formation of the tubular ER-protrusions, it is tempting to speculate about a role of this predicted enzymatic function in the membrane shaping activity of NS1-2. However, the functional significance of the ability of GII.4 NS1-2 to form filamentous ER tubules still remains unclear, since MNV NS1/2 is devoid of this property. Overall these data are suggestive for the presence of an enzymatic activity residing in NS1-2, but they clearly provide no formal proof, which will require the demonstration of such activity in a purified protein. Interestingly, a recent study identified an interaction of the variable, unstructured N-terminus of MNV NS1/2 with the host proteins VAPA and VAPB, critical for viral replication [64]. VAPA has already been observed to complex with Norwalk NS1-2 [65], presumably via a different, yet to be defined region. More strikingly, VAPA and VAPB have been implicated in the biogenesis of the HCV replication compartment via interaction with NS5A (reviewed in [23]). Therefore, VAPA and VAPB interactions might contribute to the function of NS1-2 in the formation of the norovirus replication organelle, or even be a common host factor of many DMV-type positive strand RNA viruses.

Overall, our data provide a first step to define a role for GII.4 NS1-2 in the biogenesis of the huNoV replication compartment in proliferating membranes of the smooth ER, thereby generating the material that could be transformed into vesicle clusters by other NS-proteins and suggesting a role of the predicted hydrolase function in this process.

Our study demonstrates that NS3 and NS4 both had the ability to create organized ER structures known as convoluted membranes, tubulo-reticular structures, crystalloid ER or cubic membranes, which have been found in a variety of viral infections and upon overexpression of ER membrane shaping proteins (reviewed in [58,59]). Interestingly, similar structures have been found upon expression of HAV 2C and 2BC, with the former regarded as the functional counterpart of norovirus NS3 in picornaviruses [34]. These structures clearly resemble OSER membranes with cubic (NS3) and hexagonal (NS4) symmetry. It has been reported that OSER structures can be generated through weak protein-protein interactions, which can even be triggered by overexpression of GFP tagged to cytochrome b(5), with the GFP moiety providing the homotypic interactions [66]. Therefore, we currently cannot exclude that the fusion of NS3 and NS4 with eGFP, required for CLEM, also contributed to this phenotype. However, it seems unlikely that these structures are essential for viral replication, since they have not been observed so far in either MNV infected cells or upon ORF1 overexpression [15,44]. Therefore, the ability of NS3 and NS4 to induce cubic membranes might point to an intrinsic property, probably weak homotypic interactions, and seems to be linked to strong overexpression of single NS proteins. Upon ORF1 expression and virus infection, such structures might be prevented due to the interactions among NS-proteins and by lower local concentrations of individual NS-proteins. Also in the case of HAV, cubic membranes were only found upon 2C/2BC overexpression [34] but not in HAV infected cells [28].

The strong association of NS3 with ER membranes surrounding LDs as well as its potential to induce convoluted membranes suggests an active function in the formation of GII.4 replication organelles, which clearly requires more detailed studies beyond the scope of this manuscript. A similar localization pattern was previously found for MNV NS3 in Vero cells, but not tested for LD localization at that point [13]. Interestingly, NS1-2 co-localized to NS3 on LDs in most cells upon expression of ORF1, whereas this LD association was never found upon sole expression of GII.4 NS1-2. This result argues for an additional role of NS3 in recruiting NS1-2 upon formation of the viral replication organelle, probably involving direct interactions. The recently observed localization of MNV NS3 with microtubules and cholesterol rich lipids when transiently expressed in Vero cells [45,46] further argues for a function of NS3 in shaping the replication organelle. Whether or not the NTPase activity, which has been demonstrated for the NS3 protein of GI.1 [14], is important for localization and membrane activity of NS3 remains to be determined. However, the most obvious putative function of NS3 in RNA replication is a supposed helicase activity, suggested by conserved SF3 helicase motifs [67]. Still to date, only the related 2C protein from the picornavirus *Enterovirus* 71 has been shown to function as an ATP dependent helicase [68].

NS4 is by far the most enigmatic among the norovirus NS proteins and little is currently known about its structure or function. Here, we provide the first direct experimental evidence suggesting that NS4 indeed might be the central organizer of the norovirus replication complex. In contrast to HCV, where all NS proteins can induce the formation of vesicular structures [31], NS4 was, in our hands, the only GII.4 protein capable of vesicle formation upon individual expression. In addition, SMVs and DMVs were found, similar to MNV infection and ORF1 expression, albeit with differing abundance. Altogether, these data suggest that NS4 may be the key driver in the formation of norovirus induced replication vesicles, requiring auxiliary functions of NS1-2 and NS3 to finally shape the replication organelles. This is reminiscent of HCV, where the complexity of the so called membranous web is only found upon expression of a polyprotein precursor encoding NS3 to NS5B, arguing for a concerted action engaging several NS-proteins [31,33]. In the case of picornaviruses, 2BC [27], 2BC/3A [26], 2C [34,69] and 3AB [35] have been found to generate distinct membrane alterations upon individual expression, with 2BC/3A and 3AB generating DMVs. However the ultrastructure of the replication organelles is far more complex upon infection also in case of picornaviruses [55]. Still, it is interesting to note that the unrelated proteins NS5A of HCV and 3A of PV are capable of inducing DMVs and share a similar structural organization. Specifically, both contain a unique structured region lacking enzymatic functions, which has been resolved for HCV NS5A [70] and PV 3A [71], an intrinsically unfolded region engaged in recruitment of host factors [72–74] and a membrane attachment region. Our current prediction for the organization of NS4, albeit highly speculative at this point, is strikingly similar regarding subdomain organization and functions (Fig 8A). This model will provide a valuable starting point for further in depth studies regarding the function of norovirus NS4.

Another important aspect that needs to be addressed in future studies is the function of NS4 as part of various stable polyprotein cleavage intermediates. Our study, including three different GII.4 strains suggests a delayed cleavage of the NS4-NS7 precursor, based on *in vitro* translation. However, a far more detailed analysis of polyprotein cleavage kinetics using a different GII.4 strain suggests a number of cleavage intermediates with NS4, including NS4-NS7, NS4-NS6 and NS4-NS5 [52], which might serve specific functions in the norovirus replication cycle. It is particularly tempting to speculate that delayed cleavage of NS4-NS7 might avoid diffusion of the replicase components, since NS4 seems the only protein associated with membranes in this precursor protein.

Our model of the putative membrane organization of NS1-2, NS3 and NS4 (Fig 8A) is still highly speculative and contains several uncertainties regarding transmembrane topology. While our predictions are in favor of one transmembrane domain for both, the NS1-2 and NS3 proteins, this topology would require a post-cleavage membrane insertion to keep the cleavage site accessible to NS6. Alternatively, and still in line with the predictions, NS1-2 could span the membrane twice and NS3 may harbor an amphipathic alpha helix tethering the protein to membranes, thereby keeping the NS6 cleavage site in the cytoplasm. Regardless, the transmembrane topology of NS1-2 and NS3 can be experimentally addressed in future studies using our expression model and, for example, testing the accessibility of N- and C-termini to proteases in cell lysates.

In summary, our study reveals a first insight into the organization of the putative GII.4 replication organelle and the contribution of individual NS proteins to its biogenesis using a protein expression model. In the case of HCV, comparable expression models have been invaluable to study formation and structure of the viral replication organelles as they allow mechanistic studies using replication deficient mutants or inhibitors interfering with replication. Thereby, the contribution of viral NS-proteins could be clearly defined [31,33] in addition to the importance of host factors like PI4KA [75,76]. Only expression models allowed us to identify viral membrane alterations as targets of direct antiviral agents (NS5A inhibitors [37]) and host targeting drugs including PI4KA inhibitors [75] and cyclophilin inhibitors [38]. Similar approaches might help to identify novel strategies to develop drugs targeting norovirus replication in future studies. Furthermore, our study lays the groundwork for an in-depth analysis of the functions of NS1-2 and NS4 in replication complex formation.

## Material and methods

### Cell lines and viruses

All cell lines were cultured in Dulbecco’s Modified Eagle Medium (DMEM; Life Technologies, Darmstadt, Germany) supplemented with 10% fetal bovine serum, non-essential amino acids (Life Technologies, Darmstadt, Germany), 100U/ml penicillin and 100ng/ml streptomycin (Life Technologies) and cultivated at 37°C and 5% CO_2_. The human hepatoma cell line Huh7 (maintained in our laboratory), stably expressing T7 RNA polymerase under blasticidine selection (5 μg/ml, Invitrogen, Germany) [74], was used for transient expression of plasmids encoding GII.4 NoV proteins that were analyzed by immunofluorescence and Western blot assays. Huh7 cells expressing the MNV receptor CD300lf were generated by transduction with a lentiviral vector encoding the murine CD300lf cDNA [47] (generous gift from R.C. Orchard and H.W. Virgin). Cells were selected by puromycin to obtain a stable culture of Huh7 cells with CD300lf expression. The murine macrophage cell line RAW 264.7 was obtained from ATCC (Middlesex, UK) and used for infection with MNV. MNV-CW1 [44] was used at a multiplicity of infection of 1 and analyzed 24 h after infection, unless otherwise stated. HEK293T-cells (Birke Bartosch, Lyon) were used for production of MNV virus stocks upon transfection of plasmid pMNV-CW1 (generous gift from H.W. Virgin).

### Plasmid constructs

The genomes representing consensus sequences of respective patient isolates of three GII.4 strains including a Den Haag 2006b variant (DH) (GenBank accession no. AB447456), a New Orleans 2009 variant (NO) (GenBank accession no JQ613573) and a Sydney 2012 variant (Syd) (GenBank accession no JX459908) were used in this study. Coding sequences corresponding to ORF1 of the three isolates were synthesized with protein sequences identical to the GenBank entries in vector pBMH by Biomatik (Cambridge, Canada). Full length ORF1 of NO, Saga, Sydney, MNV were amplified by PCR from the pBMH construct. Restriction sites NcoI and PacI were used to insert fragments into a basic pTM1-2, AgeI and PacI was used for insertion into basic pTM 1–3.

To generate pTM vectors allowing expression of N-terminally HA- or eGFP tagged individual norovirus nonstructural proteins, respective coding sequences were amplified using primers given in Table 1 and cloned into pTM-HA or pTM-eGFP, respectively using the indicated restriction sites.

**Table 1:** Primer sequences used for cloning.

Construct	Primer	Sequence	Enzyme
pTMeGFP ORF1 NewO.	sense	caagaccggtatgaagatggcgtctaa	AgeI
	antisense	acttaattaattactcgacgccatcttcattca	PacI
pTMHA ORF1 NewO.	sense	aataccatggcaaagatggcgtctaacg	NcoI
	antisense	cttaattaattactcgacgccatc	PacI
pTMeGFP NS1-2	sense	aagaccggtatgaagatggc	AgeI
	antisense	cactagtacgcgtttactgtagttcaaattg	SpeI
pTM eGFP NS3	sense	aagaccggtatgggacctgaggatcttgcg	AgeI
	antisense	ccactagtacgcgtttactgtagttcaaattc	SpeI
pTMeGFP NS4	sense	agaccggtatgggcccagctctcaccaccttc	AgeI
	antisense	ccactagtacgcgtttactcagttttgatgtcg	SpeI
pTMeGFP NS5	sense	aagaccggtatgggtaagaaaggg	AgeI
	antisense	ccactagtacgcgtttactcaaaactgagtttctc	SpeI
pTMeGFP NS6	sense	aagaccggtatggccccaccaagcatctgg	AgeI
	antisense	ccactagtacgcgtttattcaagtgtggcc	SpeI
pTM eGFP NS7	sense	cgtccaggagcgcaccatcttct	PfoI
	antisense	gatggcgtcgagtaaacgcgtactagtg	SpeI
pTM HA NS1-2	sense	gctaccggtatgaagatggcgtctaacgacgcttc	AgeI
	antisense	cactagtacgcgtttactgtagttcaaattgtag	SpeI
pTM HA NS3	sense	taccggtatgggacctgaggatcttgcggtgg	AgeI
	antisense	ccactagtacgcgtttactgtagttcaaatt	SpeI
pTM HA NS4	sense	gctaccggtatgggcccagctctcacc	AgeI
	antisense	actagtacgcgtttactcagttttgatg	SpeI
pTM HA NS5	sense	gctaccggtatgggtaagaaaggg	AgeI
	antisense	ccactagtacgcgtttactcaaaactgagtttc	SpeI
pTM HA NS6	sense	ctaccggtatggccccaccaagcatctggtcg	AgeI
	antisense	cactagtacgcgtttattcaagtg	SpeI
pTM HA NS7	sense	gctaccggtatgggtggtgacaacaaggggac	AgeI
	antisense	cactagtacgcgtttactcgacgccatcttc	SpeI
pTM ORF1 NewO:	sense	gtaccggtatgaagatggcgtctaacgacgcttccgctgcc	AgeI
	antisense	gacttaattaattactcgacgccatcttcattcacaaaactgg	PacI
pTM ORF1 Sydney	sense	taccatggatgaagatggcgtctaacgacgcttccgc	NcoI
	antisense	acttaattaatcactcgacgccatcttcattcacaaaactgggagccaga	PacI
pTM ORF1 DenHaag	sense	aataccatggatgaagatggcgtctaacgacgctt	NcoI
	antisense	gacttaattaattattcgacgccatcttcattcac	PacI
pTM ORF1 MNV	sense 1	taataccatggatgaggatggcaacgccatct	NcoI
	antisense 1	gtcaaagagctcagcaagcaagatcag	SacI
	sense2	ttgctgagctctttgacatcttttggaccc	SacI
	antisense2	tccactagtttactcatcctcattcacaaagactgc	SpeI
pTM eGFP NS1-2 MNV	sense	aagaccggtatgaggatggcaacgccatcttc	AgeI
	antisense	ccactagtacgcgtttattcggcctgccattccccgaagata	SpeI
pTM eGFP NS1-2 H139 NewO.	sense 1	aagaccggtatgaagatggc	AgeI
	antisense 1	cctagtacaagacctcgctccacatacaggccataagcgtagatttccccgtcc	
	sense 2	ggacggggaaatctacgcttatggcctgtatgtggagcgaggt	
	antisense 2	Cactagtacgcgtttactgtagttcaaattg	SpeI
pTM eGFP NS1-2 C205 New O.	sense A1	Aagaccggtatgaagatggc	AgeI
	antisense A1	ggtccaggacccagcaacaaaaggcataagcgttgttgtcaaaggctg	
	sense A2	cagcctttgacaacaacgcttatgccttttgttgctgggtcctggacc	
	antisense A2	Cactagtacgcgtttactgtagttcaaattg	SpeI
CMV-MNV- H150A	sense 1	Aaattaattacatgacccc	AseI
	antisense 1	cgatgtagacagagtaagcgtaaaacttgtgatcatcctg	
	sense 2	caggatgatcacaagttttacgcttactctgtctacatcg	
	antisense 2	Aaagagctcagcaagcaagatcagggcattgac	SacI
CMV-MNV-C216A	sense 1	Aaattaattacatgacccc	AseI
	antisense 1	ccagcagcagacctgataagcgttgaccggtggtggccacgtag	
	sense 2	ccaccaccgtcaacgcttatcaggtctgctgctggatt	
	antisense 2	Aaagagctcagcaagcaagatcagggcattgac	SacI

All PCR amplifications for cloning were performed with Phusion Flash High-Fidelity PCR Master Mix according to the manufacturer’s instructions (Thermo Fisher Scientific, Germany). PCR products were separated by agarose gel electrophoresis and purified with NucleoSpin Gel and PCR Clean-up kits purchased from Macherey-Nagel (Germany). Restriction digests were performed according to the instructions of the manufacturer (New England Biolabs). All parts of plasmid sequences amplified by PCR were analyzed by Sanger-sequencing to verify sequence fidelity and the correct reading frame (GATC Biotech, Konstanz, Germany).

### Production of infectious MNV virus stocks

MNV stocks were obtained by transfecting plasmid pMNV-CW1 (generous gift from Herbert W. Virgin) into 293T cells, as described [77]. 293T cells were seeded in 10 cm tissue culture dish (Corning, Durham, NC, USA) at a density of 3x10^5^ cells/ml in 15.5 ml of complete DMEM. After 24 hours, cells were transfected with 15 μg of pMNV-CW1 plasmid DNA using TransIT-LT1 Transfection Reagent (Mirrus Bio LLC, Madison, WI, USA) following the instructions of the manufacturer. Transfections were incubated for 48 hours. Virus stocks were obtained by harvesting the cells in their culture medium and twice freezing at -80°C and then thawing at 37°C. Lysates were then centrifuged at 2500 x g for 5 minutes and clarified virus stocks were stored at -80°C. To determine virus titers, RAW 264.7 cells were seeded in 96 well tissue culture plates (Corning, Durham, NC, USA) at a density of 2x10^4^ cells per well. After 24 hours, wells were infected in quadruplicate with serial dilutions of virus stocks diluted in DMEM medium. Assays were harvested 72 hours post infection by aspirating supernatant, washing with PBS and staining with 50 μl of a 1.25% (w/v) crystal violet solution (Merck, Darmstadt, Germany) in a 25% (v/v) ethanol solution for 10 minutes at room temperature. The wells were washed twice with distilled water and scored as positively infected or negative. The TCID50/ml was then calculated using the Spaerman-Kärber method [78,79].

### Immunofluorescence (IF) microscopy

For the transient expression of norovirus eGFP or HA-tagged proteins, Huh7 T7 cells were transfected with LT1 transfection agent (Mirus Bio LLC, Madison, WI, USA) according to the manufacturer's instructions. Cells were processed for IF as described in [76]. Briefly, cells were fixed in 4% paraformaldehyde (PFA) for 20 min and permeabilized with 0.5% Triton X-100 (PBS) for 15 min for co-localization analysis of eGFP-tagged proteins with subcellular markers. Primary antibodies were incubated in 3% bovine serum albumin (BSA) for 1 h at room temperature (RT). NS3 was detected with an in-house created rabbit polyclonal serum, animals were immunized with NS3 expressed and purified from E. coli by Davids Biotechnology, Regensburg, Germany. Antibodies detecting NS4/p20 and NS6 of GII.4 were a generous gift from Stefan Taube [80]. Anti NS5/VPg (strain SAB60) and anti NS7 (NO-strain) sera were raised in rabbits (Eurogentech) based on purified proteins expressed in E. coli. αNS3_MNV_ PAB was kindly provided by Prof. Dr. Ian Goodfellow, Cambridge University, UK, and was obtained by immunization of rabbits with GV MNV and GI sequences [81]. Subcellular compartments and dsRNA were labeled by the following commercially available antibodies: SEC31A/ COPII Vesicles: BD Bioscience / 612351 (Becton Dickinson GmbH, NJ USA); Golgi Apparatus: Anti-Golgin 97 (ab84340, Abcam); autophagosome: p62/ SQSTM1 (M162-3, MBL Life science). Mitochondria were labeled with MitoTracker Deep Red (M22426) and LDs were stained with HCS LipidTox Red Neutral Lipid Stain (Thermo Fisher Scientific Waltham, Massachusetts, USA). ER was stained with a polyclonal anti-PDI antibody (ab31811; Abcam, Cambridge, UK) unless otherwise stated. In case co-staining did not allow the use of this antibody, a mouse monoclonal antibody against Climp-63 (mainly rough ER, ALX-804-604, Enzo) or reticulon 3 (RTN3 sc-374599, Santa Cruz Biotechnology) was used. The lysosome was marked with anti LBPA1 antibody (Clone 6C4, Sigma-Aldrich, Germany). All primary antibodies were utilized in a 1:50 dilution. Alexa 488 or 647-conjugated secondary antibodies (Invitrogen, Molecular Probes) were incubated in 3% BSA for 45 min at RT with a dilution of 1:1000. Nuclei were stained using 4,6-diamidino-2-phenylindole (DAPI) for 1 min at a dilution of 1:4000 after incubation with secondary antibodies. Cells were mounted with Fluoromount G (Southern Biotechnology Associates, Birmingham, AL, USA). Confocal microscopy was conducted on a Leica SP5 AOBS Point Scanning Confocal Microscope (Leica Microsystems). Confocal microscopy was conducted on a Leica SP5 and on Leica SP8 AOBS Point Scanning Confocal Microscopes (Leica Microsystems). Image analysis was performed using the ImageJ software package Fiji (http://fiji.sc/wiki/index.php/Fiji) [82] and the Coloc 2 plugin was used to calculate the Pearson's correlation coefficient. A Pearson's correlation coefficient higher than 0.5 indicates a strong colocalization.

### Conventional preparation of cells for electron microscopy (EM)

Cells grown on glass coverslips were subjected to chemical fixation and subsequent epon embedding. For overexpression of norovirus proteins, Huh7-Lunet T7 cells were transfected with TransIT-LT1 (Mirus, Madison USA) transfection reagent according to manufacturer’s instructions and fixed 24 hours post transfection. RAW 264.7 (ATCC, UK) were used for infection with MNV-CW1 [83]. For chemical fixation, cells were washed 3 times with 1x PBS and fixed for 30 min with pre-warmed 2.5% glutaraldehyde in 50 mM sodium cacodylate buffer (pH 7.2) containing 1 M KCl, 0.1 M MgCl_2_, 0.1 M CaCl_2_ and 2% sucrose. Cells were washed thoroughly 5 times with 50 mM cacodylate buffer and post-fixed on ice in the dark with 2% OsO_4_ in 50 mM cacodylate buffer for 40 min. Cells were washed with H_2_O overnight, treated with 0.5% uranyl acetate in H_2_O for 30 min, rinsed thoroughly with H_2_O and dehydrated in a graded ethanol series at RT (40%, 50%, 60%, 70% and 80%) for 5 min each and 95% and 100% for 20 min each. Cells were immersed in 100% propilene oxid and immediately embedded in an Araldite-Epon mixture (Araldite 502/Embed 812 Kit; Electron Microscopy Sciences). After polymerization at 60°C for 2 days, coverslips were removed and the embedded cell monolayers were sectioned using a Leica Ultracut UCT microtome and a diamond knife. Sections with a thickness of 70 nm were counter-stained with 3% uranyl acetate in 70% methanol for 5 min and 2% lead citrate in H_2_O for 2 min, and examined with the transmission electron microscope Philips CM120 TEM (Biotwin, 120 kV).

### Correlative light-electron microscopy (CLEM)

Huh7-T7 cells seeded on glass-bottom dishes containing a photoetched gridded coverslip (MatTek) were transiently transfected with expression plasmids coding for GFP-fused norovirus proteins. After 24 h cells were washed twice with PBS, fixed for 30 min at room temperature with PBS containing 4% paraformaldehyde and 0.2% glutaraldehyde and stained with DAPI and far-red LipidTOX neutral lipid stain (Thermofisher) according to the manufacturer's instructions. Samples were analyzed on a Nikon TE2000 Ultraview ERS spinning disc (PerkinElmer). Z-stacks of GFP-positive cells were collected and the positions of the cells of interest were recorded using transmitted light with a differential interference contrast configuration. Cells were then fixed in EM fixative and embedded in Epon/araldite resin, as described above.

Seventy nm ultrathin sections were prepared and examined with a Jeol JEM-1400 transmission electron microscope (Jeol Ltd., Tokyo, Japan). Landmark correspondence plugin from Fiji imageJ distribution was used to correlate the light microscopy and the electron microscopy datasets. Briefly, a single optical section displaying a LDs distribution that matched the one observed in the electron microscopy micrograph was extracted from the z-stack and the corresponding LDs on the two images were used as landmark to calculate the transformation.

### High pressure freezing and freeze substitution (HPF-FS)

Cells were seeded onto 3 mm sapphire discs (M. Wohlwend GmbH, Sennwald, Switzerland) that had been carbon coated to improve cell adhesion. One day after transfection or infection cells (Huh7-Lunet T7 and RAW 264.7, respectively) were frozen after immersion in 1-hexadecene (Merck, Hohenbrunn, Germany) using a high-pressure freezer (M. Wohlwend GmbH). Frozen discs were stored in liquid nitrogen until further processing. Freeze substitution was done in acetone containing 0.2% (w/v) OsO_4_, 0.1% (w/v) UA, and 5% (v/v) water by slowly warming the samples from −90°C to 0°C during a period of 20 h [84]. Samples were kept at 0°C and at room temperature for 30 min each, washed with acetone, and embedded in four-step epon series (Fluka, Buchs, Switzerland) using 1 h-incubation in 25%, 50% and 75% epon dissolved in acetone and overnight incubation in 100% epon. Epon was exchanged, polymerized for 3 d at 60°C and sapphire discs were removed by immersion in liquid nitrogen. Seventy or 250 nm thick sections for were examined by conventional transmission EM or electron tomography, respectively.

### Electron tomography (ET)

Sections of 250 nm thickness were collected on palladium-copper slot grids (Science Services, Munich, Germany) coated with Formvar (Plano, Wetzlar, Germany). Protein A-gold (10 nm) was added to both sides of the sections as fiducial markers. Single axis tilt series were acquired with a FEI TECNAI F30 microscope operated at 300 kV and equipped with a 4k FEI Eagle camera over a −65° to 65° tilt range (increment 1°) and at an average defocus of −0.2 μm. Reconstruction of the tomograms and rendering of their 3D surface was performed by using the IMOD software package (version 4.9)[85] (bio3d.colorado.edu/imod).

### Western blot (WB)

For Western blotting, the cells in a 6-well plate well were lysed and denatured in 150 μL of 6× Laemmli buffer by heating to 95°C for 5 minutes, and loaded onto an 12% polyacrylamide-SDS gel. After resolution by SDS-PAGE, proteins were transferred to a polyvinylidene difluoride (PVDF) membrane, with the exception of NS7, which was transferred to a nitrocellulose membrane (Amersham Protran 0.45 NC, GE Healthcare Life Science). NS proteins were detected using NS-protein specific polyclonal rabbit antibodies described in IF section in a 1:1000 dilution. β-actin was detected by monoclonal mouse antibody (A5441), Sigma-Aldrich. Primary antibodies were detected using αrabbit/ αmouse horseradish peroxidase (HRP)-coupled secondary antibodies (Sigma-Aldrich) and imaging was done with the ChemoCam 6.0 ECL system (INTAS Science Imaging, Goettingen, Germany).

### Coupled *in vitro* transcription-translation assay

pTM based constructs were phenol/chloroform purified and reconstituted in RNase-free double-distilled H_2_O to a concentration of 1 μg/μL. 0.5 μg of the plasmid preparation were then mixed with 10 μL of the L1170 T7 TNT-kit (Promega, Madison, USA) and 1μL of ^35^S Methionine 10 mCi/ml. The reaction mixture was then incubated at 30°C for 90 min. Afterwards, the reaction was suspended in 2x laemmli buffer, and denatured at 95°C for 5 min before being loaded onto a 12% SDS gel for electrophoresis. Radiolabeled proteins were visualized by autoradiography using a phospho-imager (BioRad, Munich, Germany).

### Generation of Huh7 cells expressing the MNV receptor CD300lf

Stable cell lines expressing the MNV receptor CD300lf [47,48], were created by lentiviral transduction. The lentiviral vectors were created by co-transfecting 293T cells with a gag-pol plasmid (pCMV∆8.31), the retroviral vector containing the CD300lf sequence (gift from Dr. Herbert Virgin, Washington University at St. Louis, St. Louis, MO, USA), and an envelope plasmid (pMD.G) mixed in a 3:3:1 ratio, respectively, as described elsewhere [86]. Huh7 cells were seeded at a density of 1x10^5^ cells per well in 6-well plates and transduced with 1 ml of lentivirus suspension mixed with 1 ml of fresh DMEM medium for 12 hours. Media was aspirated and replaced with 1:1 lentivirus suspension and 1 ml of fresh DMEM two more times at 12 hour intervals. After 36 hours, the media was changed and 2 ml of fresh DMEM containing 2 μg/ml puromycin selection was added and selective pressure was maintained during passaging.

### Modelling of the structure of NS1-2, NS3 and NS4

We used resources of the MPI bioinformatics Toolkit [87] to define domains in NS1-2, NS3 and NS4. HHPRED was used to find domains with homologs of known structure and Quick2D to identify structured and non-structured regions and putative transmembrane helices [88,89]. Transmembrane helices were further sought with Polyphobius. For putative membrane-peripheral and transmembrane helices, ideal alpha-helices were generated with the “*fab*” function of PyMOL, as were intervening loops. The NS1-2 central domain (residues 119 to 213) was homology modelled with SwissModel [90] from Protein Data Bank entry 4DPZ [61]. The non-structured N-terminus and helical C-terminus were then generated with PyMOL 1.8.2.0 [91]. The three parts were assembled in PyMOL with the PyMOL sculpting function. NS3 was modelled with I-Tasser [92] and NS4 was modelled with RaptorX [93], with default parameters. A membrane model was generated with the Charmm-GUI webserver [94] with a lipid composition similar to the endoplasmic reticulum (Phosphatidylcholine 60%; Phosphatidylethanolamine 25%; Phosphatidylinositol 15%)[95]. The positions of the transmembrane/peripheral helices relative to this membrane were adjusted with PyMOL. The structures were then minimized with secondary structure restraints with the Phenix geometry minimization function [96].

### Statistical analyses

General statistical analyses as indicated in the corresponding figures were performed using Graphpad Prism Software.

## Supporting information

S1 Fig MNV replication in Huh7 cells expressing the MNV receptor CD300lf. Huh7-CD300lf cells were infected (A) or mock infected (B) with MNV for 24h (MOI = 1). Cells were fixed with 4% PFA at the indicated time points post infection, permeabilized with 0.5% Triton X-100 and stained with αNS3 (red) and DAPI (blue). (C) Huh7, Huh7-CD300lf or RAW264.7 cells were infected with MNV (MOI = 1) and harvested at the indicated time points post infection in their culture medium. Clarified lysates obtained by twice freezing and thawing were titrated on RAW 264.7 cells by TCID50 assay and titers were calculated using the Spearman-Karber method. Mean and SD from a representative experiment (n = 2). Note that low titers obtained from infection of Huh7 cells are most likely due to remaining virus inoculum.
(TIF)

S2 Fig Ultrastructural changes in RAW264.7 cells upon infection with MNV. RAW264.7 cells infected with MNV at an MOI of 5 (A, B) or mock infected (C) were fixed and prepared for electron microscopy analysis 20 hours after infection, using chemical fixation (B, C) or high pressure freezing (A). Magnified views of the boxed areas are shown to the right. Single membrane vesicle (SMV), double membrane vesicle (DMV), multi membrane vesicle (MMV) and rough ER (rER) are indicated. ALS: Autophagosome-like structure. ALSs were defined as rounded organelles having two lipid bilayers that separate them from the cytosol with a size above 300 nm. Since there is no formal criterion to discriminate DMVs and ALSs apart from the size, we cannot rule out that ALSs are larger DMVs or that DMVs are small ALSs, respectively.
(TIF)

S3 Fig Characterization of GII.4 ORF1 expression *in vitro* and in Huh7-T7 cells. (A) Expression of HA-tagged individual NS-proteins (NO strain) and ORF1 from three GII.4 strains in a coupled in-vitro transcription/translation rabbit reticulocyte lysate (RRL) system in the presence of [^35^S]-Methionine for 1 hour. Proteins were separated using a 12% polyacrylamide gel and detected by phosphoimaging. (B-F) ORF1 from three GII.4 strains and individual eGFP-tagged NS-proteins from the NO-strain as indicated were expressed in Huh7-T7 cells, lysates were harvested 20 hours post transfection and analyzed by Western blotting (WB) using antibodies specific for NS3 (B), NS7 (C), NS4 (D), NS5 (E) or NS6 (F). (G) Detection of eGFP-NS1-2 after expression of ORF1 of strain NO, N-terminally tagged with eGFP (NO eGFP-ORF1) in Huh7-T7 cells, using GFP-specific antibodies. β-actin served as loading control (αAct.).
(TIF)

S4 Fig Ultrastucture of Huh7-T7 cells expressing GII.4 ORF1. Huh7 T7 cells were transfected with a pTM-based ORF1 construct of the indicated New Orleans (A), Den Haag (B) and Sydney (C) strains and processed for EM 20 hours p.t., upon CF or HPF (for further detail see the legend to Fig 2C). Magnified views of the boxed areas are shown to the right. Single membrane vesicles (SMVs), double membrane vesicles (DMVs), multi-membrane vesicles (MMVs), lipid droplets (LDs) and rough ER (rER) are indicated in the high magnification views.
(TIF)

S5 Fig Virtual xy slices from the electron tomograms shown in S1–S3 Movies. (A) MVB-DMV contact site from S1 Movie. (B) MVB-DMV contact site from S3 Movie. (C) Autophagosome-like structures (ALS) from S1 and S3 Movies. Note that we defined endosomes or MVBs as rounded organelles delimited from the cytosol by one lipid bilayer, containing a highly heterogeneous lumen composed of multiple vesicles with different sizes and electron densities. ALSs were defined as rounded organelles having two lipid bilayers that separate them from the cytosol and a diameter above 300. Their lumen, in contrast to MVBs, was only composed of cytosolic content and/or one engulfed vesicle. We cannot rule out, however, that ALSs are larger DMVs.
(TIF)

S6 Fig Integrity of N-terminally eGFP tagged individual NS proteins and localization of eGFP-NS1-2 with respect to ER and cytoskeleton markers or NS3. (A) Schematic representation of N-terminally eGFP tagged individual NS-proteins used in subsequent experiments. (B) Integrity of eGFP-tagged individual NS proteins expressed in Huh7-T7 cells. Cell lysates expressing the indicated proteins were harvested 20 hours post transfection and analyzed by Western blotting, using GFP-specific antibodies to prove the integrity of the fusion proteins. α-actin was used as loading control. (C-F) Huh7 T7 cells were transfected with N-terminally eGFP tagged NS1-2 (C, D) or eGFP-ORF1 (E, F) for 24 hours before being fixed, permeabilized and stained with antibodies specific for the indicated cellular proteins (red) (C, D) or NS3 (E, F). eGFP-NS1-2 signal is depicted in green. Scale bars 10 μm. (D, F). Quantification of the degree of co-localization calculated by Pearson's correlation coefficients between the eGFP-NS1-2 and the indicated cellular proteins (D) or NS3 (F). Each dot represents a single cell. Mean and SD are indicated. CK-8; cytokeratin 8.
(TIF)

S7 Fig Subcellular localization of eGFP-NS1/2-MNV analyzed by IF and CLEM. (A) Integrity of eGFP-NS1/2-MNV. Plasmids encoding eGFP-NS1/2-MNV or eGFP-NS1-2 were transfected into Huh7 T7 cells. Cell lysates were harvested 20 hours post transfection and analyzed by Western blotting, using GFP-specific antibodies to prove the integrity of the fusion proteins. Calnexin (αClnx) was used as loading control. (B, C) A plasmid encoding eGFP-NS1/2 MNV was transfected into Huh7 T7 cells. Twenty hours post transfection, cells were fixed and analyzed by confocal microscopy (B) or CLEM (C). (B) eGFP-NS1/2-MNV was detected by antibodies detecting GFP (green, left panel) and localization analyzed by staining of PDI as marker of the ER. (C) Cells expressing eGFP-NS1/2 were processed for CLEM. For further details see the legend to Fig 5D and M&M. Left, a 20x low magnification composite micrograph of phase contrast and fluorescence light microscopy images showing the cell of interest (yellow box) selected for electron microscopy analysis and the coordinate pattern in the background. eGFP-NS1/2 is depicted in green, LipidTox (LD) in red and nuclei in blue. Black scale bar 10 μm, white scale bar 5 μm and red scale bars 200 nm.
(TIF)

S8 Fig Subcellular localization of NS3 expressed in the context of ORF-1. A plasmid encoding the GII.4 NO polyprotein (A, C) or an empty vector (B) was transfected into Huh7-T7 cells. Twenty hours post transfection, cells were stained using NS3 specific polyclonal antibodies (green, A-C), LipidTox to stain lipid droplets (red, A, C), a reticulon-3 (RTN3) specific monoclonal antibody to stain ER membranes (red, A, C) or different monoclonal antibodies for cellular markers as indicated (C). White scale bars 10 μm, red scale bars 2 μm. (C) Quantification of the degree of co-localization calculated by Pearson's correlation coefficients between the signals of the indicated cellular markers and the NS3 signals. Each dot represents a single cell. Mean and SD are shown.
(TIF)

S1 Movie A dual-axis tomogram reconstructed from a ∼250 nm thick section of a Huh7 T7 cells transfected with pTM NO ORF1, fixed 24 hpt. Corresponding to Fig 4A, S5A Fig, S4 Movie.
(MP4)

S2 Movie A single-axis tomogram reconstructed from a ∼250 nm thick section of a Huh7 T7 cells transfected with pTM NO ORF1, fixed 24 hpt. Corresponding to Fig 4B, S5 Movie.
(MP4)

S3 Movie A dual-axis tomogram reconstructed from a ∼250 nm thick section of a Huh7 T7 cells transfected with pTM NO ORF1, fixed 24 hpt. Corresponding to S5B and S5C Fig.
(MP4)

S4 Movie Animation through a dual-axis tomogram (corresponding to Fig 4A), reconstructed from a ∼250 nm thick section of a Huh7 T7 cells transfected with pTM NO ORF1, fixed 24 hpt. Colored overlay shows a 3D surface model of GII.4 NO-induced membranes. ER membranes are depicted in dark brown, a DMV in yellow, SMVs in white, MMVs in blue and the nuclear membrane in dark green.
(MP4)

S5 Movie Animation through a single-axis tomogram (corresponding to Fig 4B), reconstructed from a ∼250 nm thick section of a Huh7 T7 cells transfected with pTM NO ORF1, fixed 24 hpt. Colored overlay shows a 3D surface model of GII.4 NO-induced membranes. SMVs are depicted in white, MMVs in blue, late endosomes in red and a microtubule in green.
(MP4)
