# Supplementary figures and images for: Membrane alterations induced by nonstructural proteins of human norovirus
Source: PLoS Pathog. 2017 Oct 27;13(10):e1006705. doi: 10.1371/journal.ppat.1006705 (PMC5678787; doi:10.1371/journal.ppat.1006705)

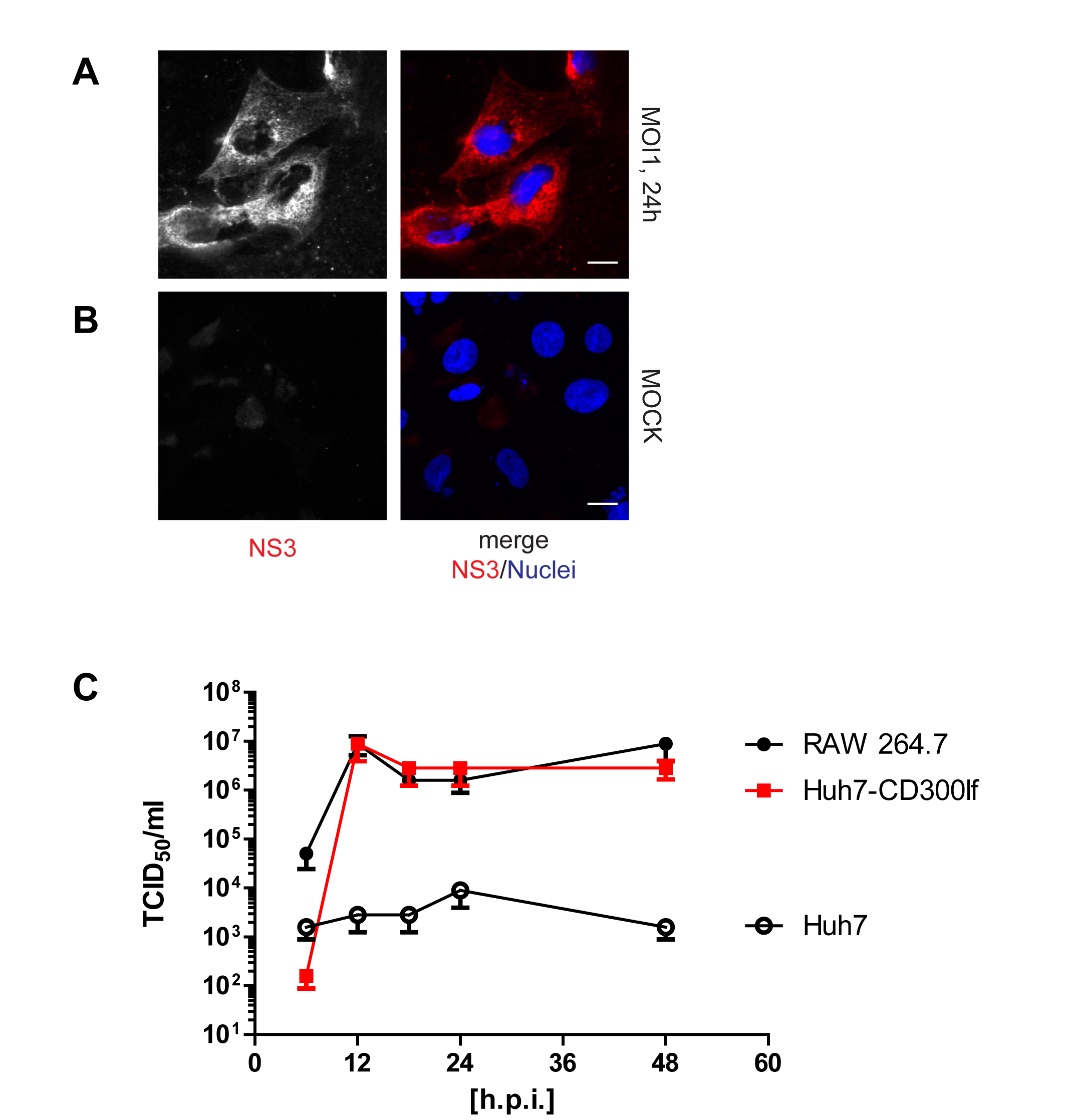

Supplement: S1 Fig — Huh7-CD300lf cells were infected (A) or mock infected (B) with MNV for 24h (MOI = 1). Cells were fixed with 4% PFA at the indicated time points post infection, permeabilized with 0.5% Triton X-100 and stained with αNS3 (red) and DAPI (blue). (C) Huh7, Huh7-CD300lf or RAW264.7 cells were infected with MNV (MOI = 1) and harvested at the indicated time points post infection in their culture medium. Clarified lysates obtained by twice freezing and thawing were titrated on RAW 264.7 cells by TCID50 assay and titers were calculated using the Spearman-Karber method. Mean and SD from a representative experiment (n = 2). Note that low titers obtained from infection of Huh7 cells are most likely due to remaining virus inoculum. (TIF) [file ppat.1006705.s001.tif]

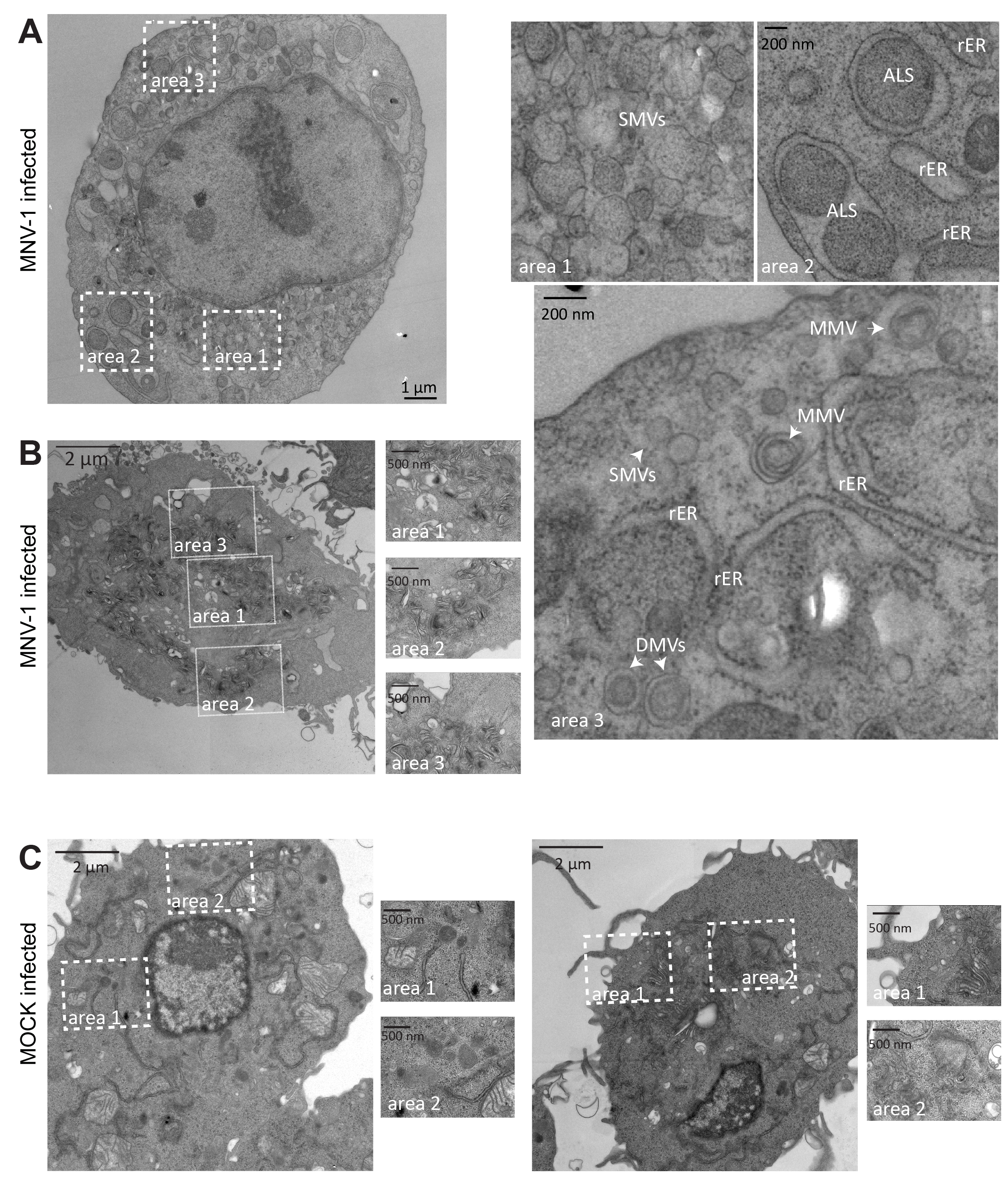

Supplement: S2 Fig — RAW264.7 cells infected with MNV at an MOI of 5 (A, B) or mock infected (C) were fixed and prepared for electron microscopy analysis 20 hours after infection, using chemical fixation (B, C) or high pressure freezing (A). Magnified views of the boxed areas are shown to the right. Single membrane vesicle (SMV), double membrane vesicle (DMV), multi membrane vesicle (MMV) and rough ER (rER) are indicated. ALS: Autophagosome-like structure. ALSs were defined as rounded organelles having two lipid bilayers that separate them from the cytosol with a size above 300 nm. Since there is no formal criterion to discriminate DMVs and ALSs apart from the size, we cannot rule out that ALSs are larger DMVs or that DMVs are small ALSs, respectively. (TIF) [file ppat.1006705.s002.tif]

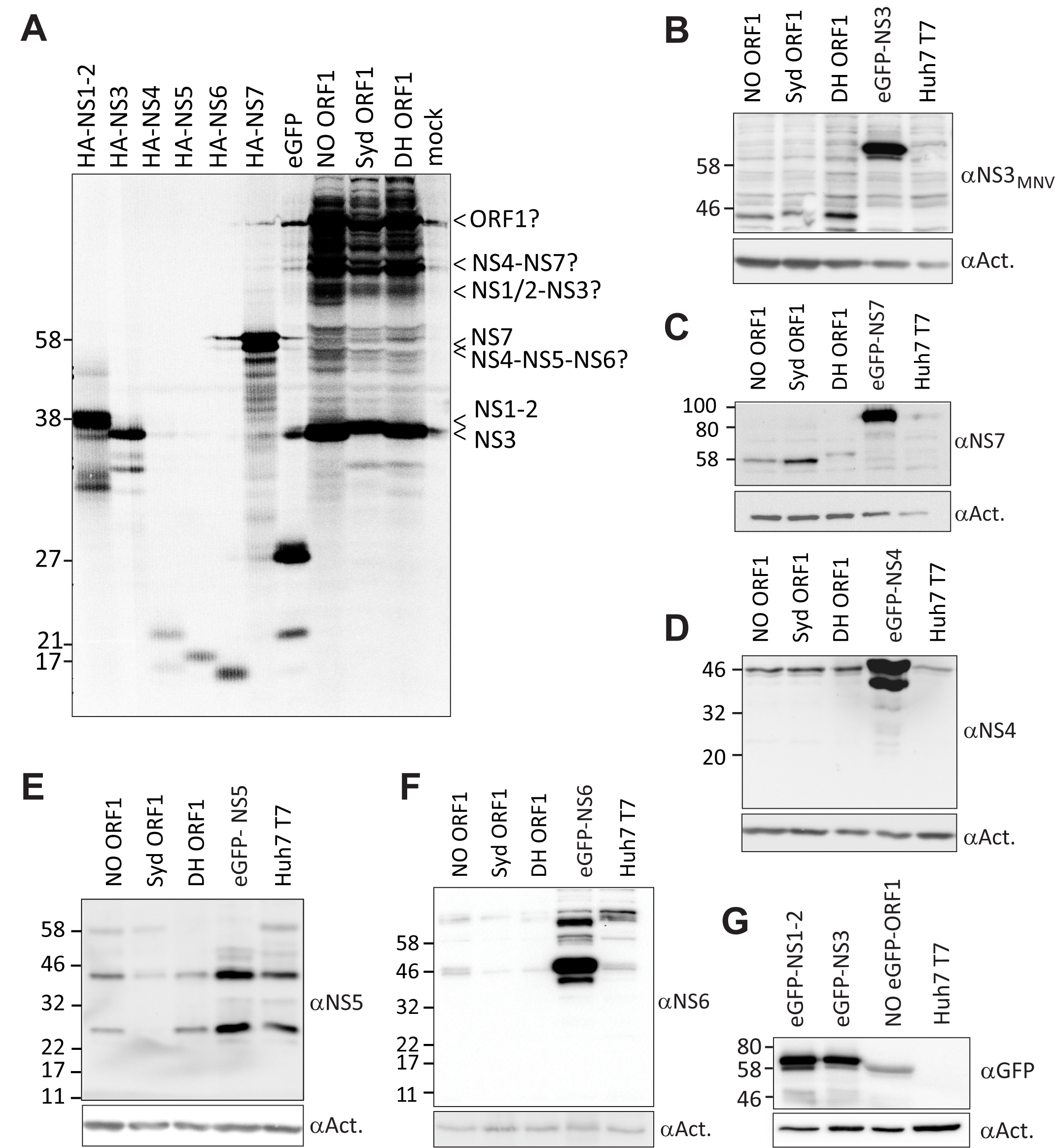

Supplement: S3 Fig — (A) Expression of HA-tagged individual NS-proteins (NO strain) and ORF1 from three GII.4 strains in a coupled in-vitro transcription/translation rabbit reticulocyte lysate (RRL) system in the presence of [35S]-Methionine for 1 hour. Proteins were separated using a 12% polyacrylamide gel and detected by phosphoimaging. (B-F) ORF1 from three GII.4 strains and individual eGFP-tagged NS-proteins from the NO-strain as indicated were expressed in Huh7-T7 cells, lysates were harvested 20 hours post transfection and analyzed by Western blotting (WB) using antibodies specific for NS3 (B), NS7 (C), NS4 (D), NS5 (E) or NS6 (F). (G) Detection of eGFP-NS1-2 after expression of ORF1 of strain NO, N-terminally tagged with eGFP (NO eGFP-ORF1) in Huh7-T7 cells, using GFP-specific antibodies. β-actin served as loading control (αAct.). (TIF) [file ppat.1006705.s003.tif]

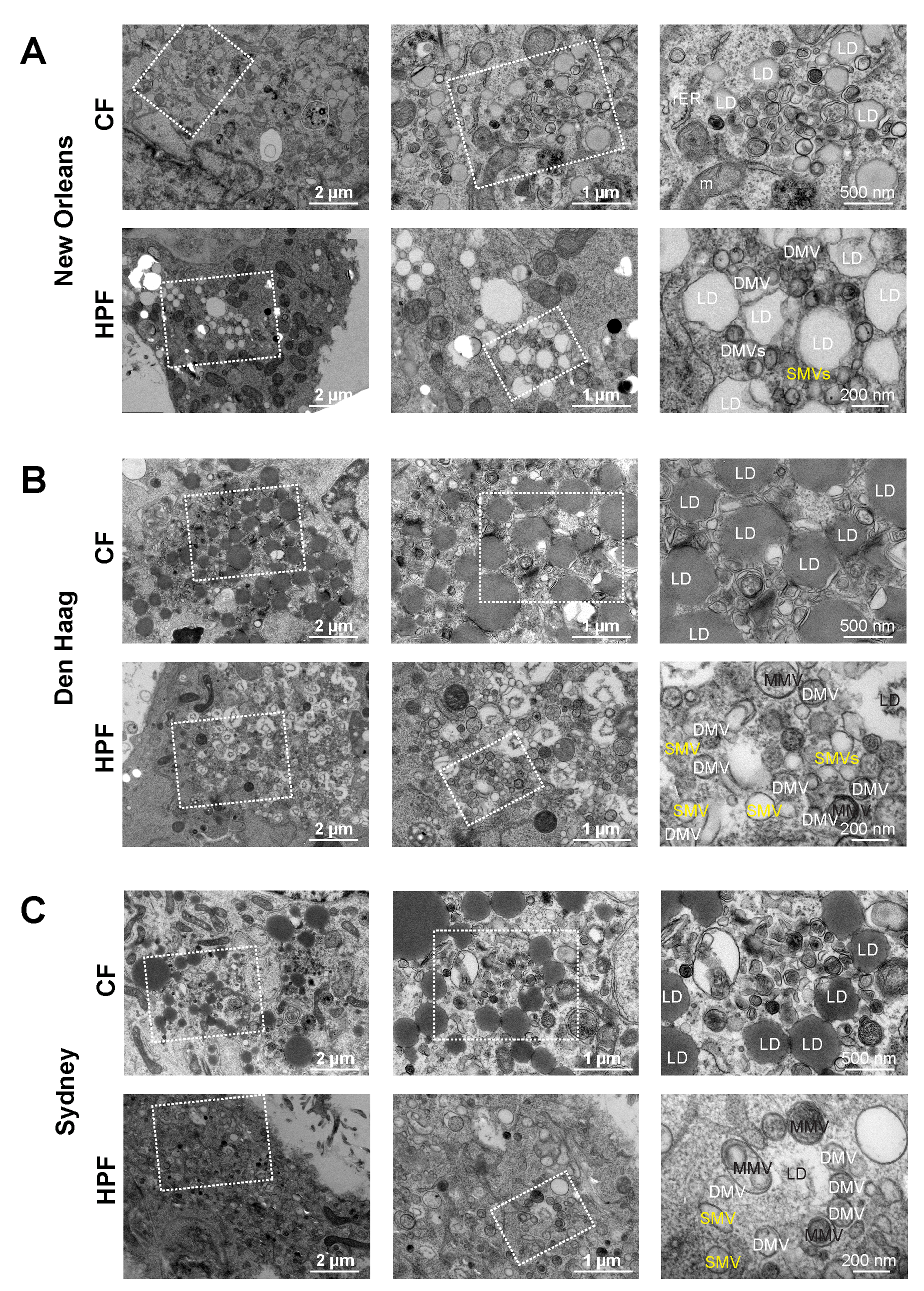

Supplement: S4 Fig — Huh7 T7 cells were transfected with a pTM-based ORF1 construct of the indicated New Orleans (A), Den Haag (B) and Sydney (C) strains and processed for EM 20 hours p.t., upon CF or HPF (for further detail see the legend to Fig 2C). Magnified views of the boxed areas are shown to the right. Single membrane vesicles (SMVs), double membrane vesicles (DMVs), multi-membrane vesicles (MMVs), lipid droplets (LDs) and rough ER (rER) are indicated in the high magnification views. (TIF) [file ppat.1006705.s004.tif]

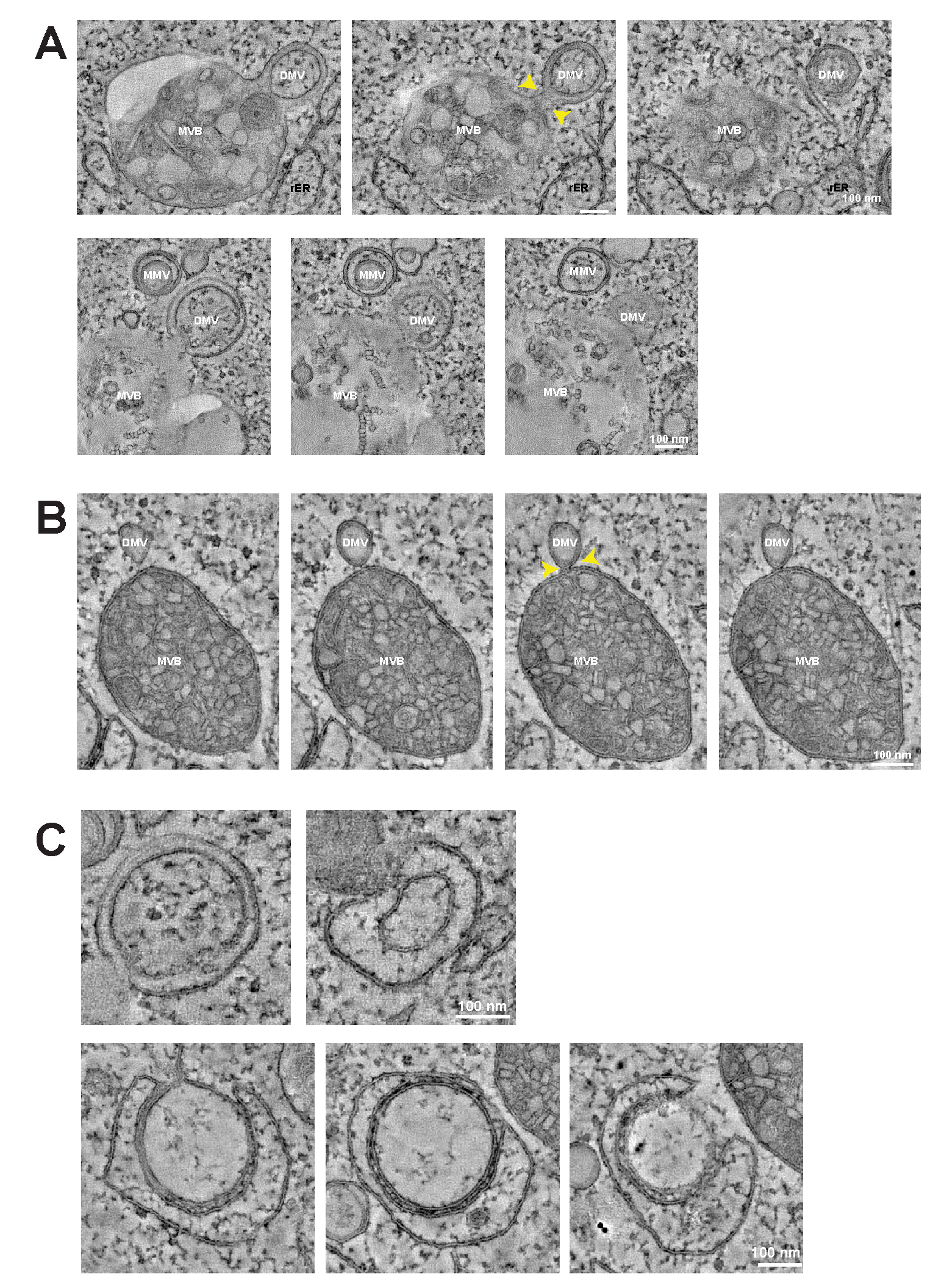

Supplement: S5 Fig — (A) MVB-DMV contact site from S1 Movie. (B) MVB-DMV contact site from S3 Movie. (C) Autophagosome-like structures (ALS) from S1 and S3 Movies. Note that we defined endosomes or MVBs as rounded organelles delimited from the cytosol by one lipid bilayer, containing a highly heterogeneous lumen composed of multiple vesicles with different sizes and electron densities. ALSs were defined as rounded organelles having two lipid bilayers that separate them from the cytosol and a diameter above 300. Their lumen, in contrast to MVBs, was only composed of cytosolic content and/or one engulfed vesicle. We cannot rule out, however, that ALSs are larger DMVs. (TIF) [file ppat.1006705.s005.tif]

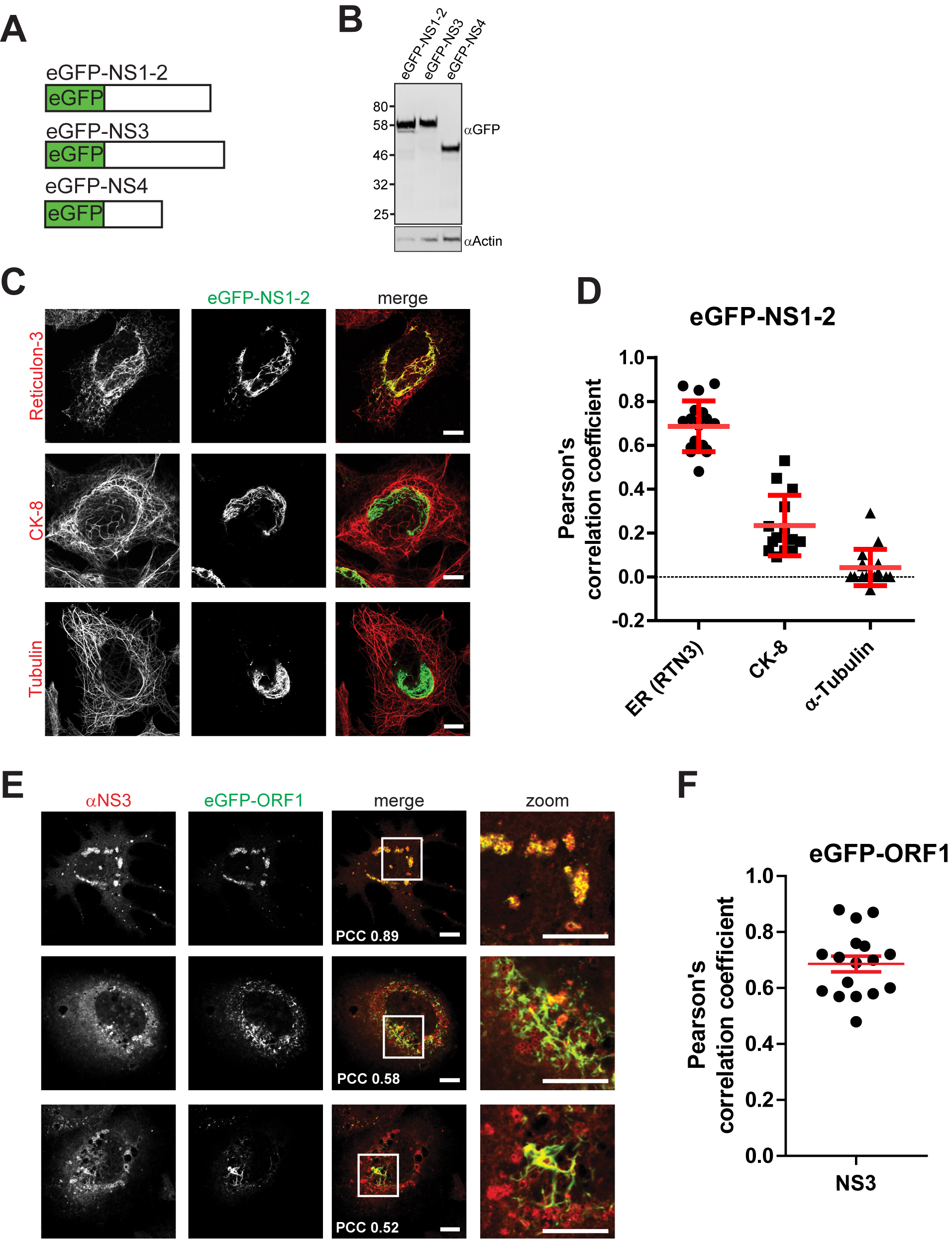

Supplement: S6 Fig — (A) Schematic representation of N-terminally eGFP tagged individual NS-proteins used in subsequent experiments. (B) Integrity of eGFP-tagged individual NS proteins expressed in Huh7-T7 cells. Cell lysates expressing the indicated proteins were harvested 20 hours post transfection and analyzed by Western blotting, using GFP-specific antibodies to prove the integrity of the fusion proteins. α-actin was used as loading control. (C-F) Huh7 T7 cells were transfected with N-terminally eGFP tagged NS1-2 (C, D) or eGFP-ORF1 (E, F) for 24 hours before being fixed, permeabilized and stained with antibodies specific for the indicated cellular proteins (red) (C, D) or NS3 (E, F). eGFP-NS1-2 signal is depicted in green. Scale bars 10 μm. (D, F). Quantification of the degree of co-localization calculated by Pearson's correlation coefficients between the eGFP-NS1-2 and the indicated cellular proteins (D) or NS3 (F). Each dot represents a single cell. Mean and SD are indicated. CK-8; cytokeratin 8. (TIF) [file ppat.1006705.s006.tif]

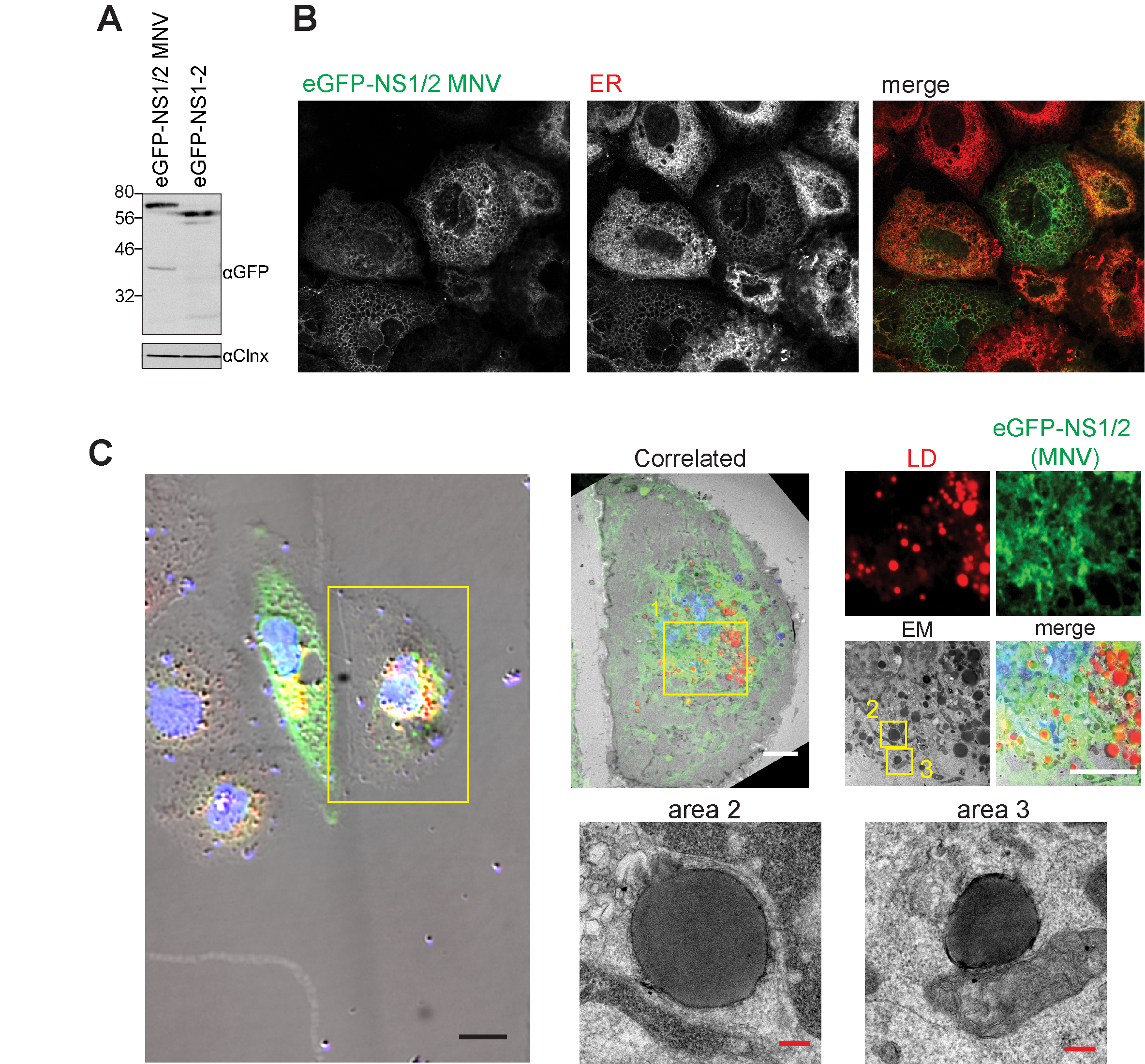

Supplement: S7 Fig — (A) Integrity of eGFP-NS1/2-MNV. Plasmids encoding eGFP-NS1/2-MNV or eGFP-NS1-2 were transfected into Huh7 T7 cells. Cell lysates were harvested 20 hours post transfection and analyzed by Western blotting, using GFP-specific antibodies to prove the integrity of the fusion proteins. Calnexin (αClnx) was used as loading control. (B, C) A plasmid encoding eGFP-NS1/2 MNV was transfected into Huh7 T7 cells. Twenty hours post transfection, cells were fixed and analyzed by confocal microscopy (B) or CLEM (C). (B) eGFP-NS1/2-MNV was detected by antibodies detecting GFP (green, left panel) and localization analyzed by staining of PDI as marker of the ER. (C) Cells expressing eGFP-NS1/2 were processed for CLEM. For further details see the legend to Fig 5D and M&M. Left, a 20x low magnification composite micrograph of phase contrast and fluorescence light microscopy images showing the cell of interest (yellow box) selected for electron microscopy analysis and the coordinate pattern in the background. eGFP-NS1/2 is depicted in green, LipidTox (LD) in red and nuclei in blue. Black scale bar 10 μm, white scale bar 5 μm and red scale bars 200 nm. (TIF) [file ppat.1006705.s007.tif]

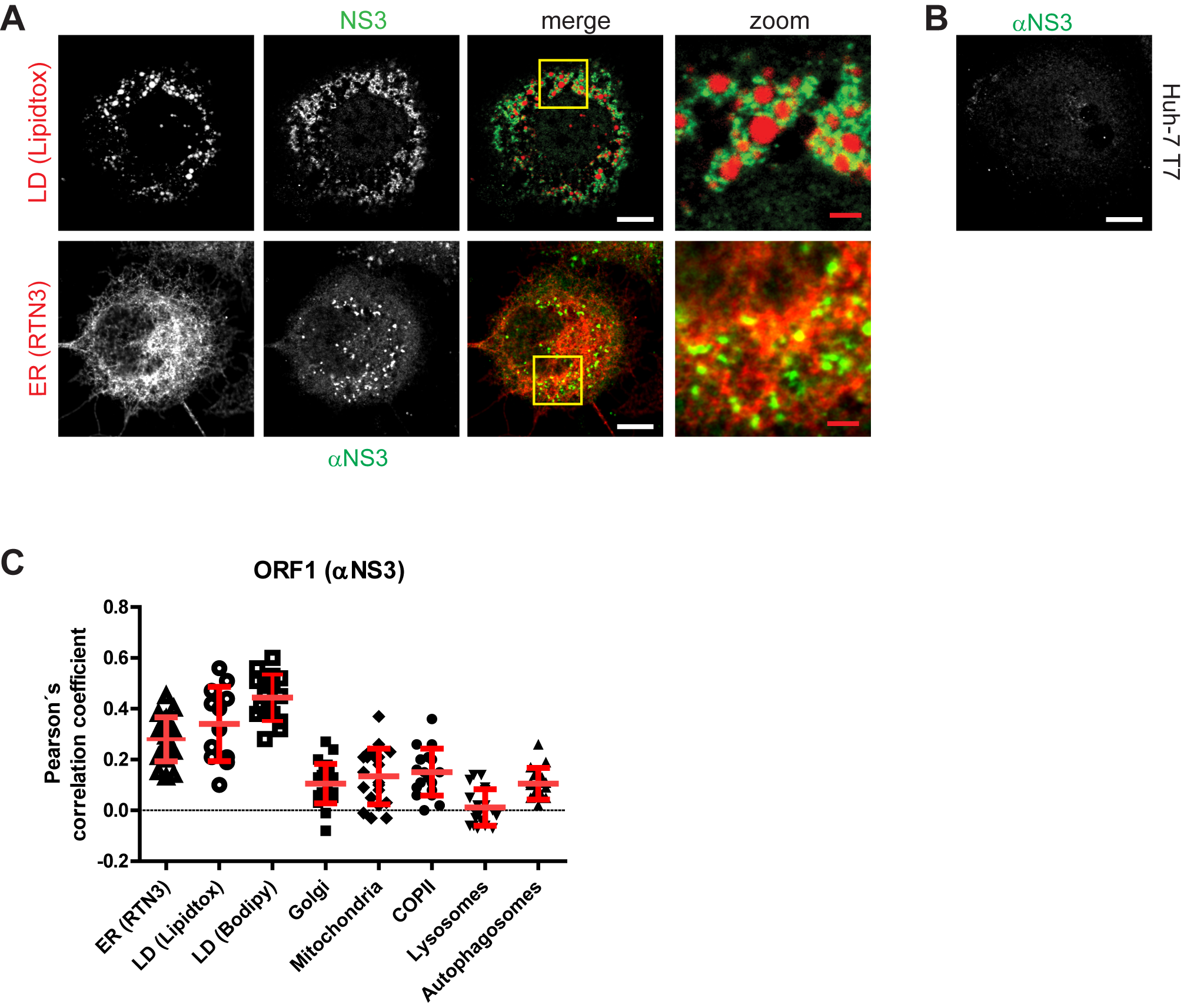

Supplement: S8 Fig — A plasmid encoding the GII.4 NO polyprotein (A, C) or an empty vector (B) was transfected into Huh7-T7 cells. Twenty hours post transfection, cells were stained using NS3 specific polyclonal antibodies (green, A-C), LipidTox to stain lipid droplets (red, A, C), a reticulon-3 (RTN3) specific monoclonal antibody to stain ER membranes (red, A, C) or different monoclonal antibodies for cellular markers as indicated (C). White scale bars 10 μm, red scale bars 2 μm. (C) Quantification of the degree of co-localization calculated by Pearson's correlation coefficients between the signals of the indicated cellular markers and the NS3 signals. Each dot represents a single cell. Mean and SD are shown. (TIF) [file ppat.1006705.s008.tif]
